# Treatment of bladder cancer by geoinspired synthetic chrysotile nanocarrier-delivered *circPRMT5* siRNA

**DOI:** 10.1186/s40824-022-00251-z

**Published:** 2022-02-05

**Authors:** Chunping Yu, Yi Zhang, Ning Wang, Wensu Wei, Ke Cao, Qun Zhang, Peiying Ma, Dan Xie, Pei Wu, Biao Liu, Jiahao Liu, Wei Xiang, Xing Hu, Xuewen Liu, Jianfei Xie, Jin Tang, Zhi Long, Long Wang, Hongliang Zeng, Jianye Liu

**Affiliations:** 1 Department of Urology, The Third Xiangya Hospital of Central South University, No.138, Tongzipo Road, Changsha, 410013 Hunan China; 2 Department of Urology, Sun Yat-sen University Cancer Center, No. 651, Dongfeng Road East, Guangzhou, 510060 Guangdong China; 3 State Key Laboratory of Oncology in Southern China, Collaborative Innovation Center for Cancer Medicine, No. 651, Dongfeng Road East, Guangzhou, 510060 Guangdong China; 4 School of Minerals Processing and Bioengineering, Central South University, No. 932, Lushan South, Changsha, 410083 Hunan China; 5 Department of Onology, The Third Xiangya Hospital of Central South University, No.138, Tongzipo Road, Changsha, 410013 Hunan China; 6 Department of Radiotherapy, The First Affiliated Hospital of Sun Yat-sen University, 58 Zhongshan 2nd Road, Guangzhou, 510080 Guangdong China; 7 Department of Operation Center, The Second Xiangya Hospital of Central South University, People’s Middle Road, Changsha, 410008 Hunan China; 8 Department of Nursing, The Third Xiangya Hospital of Central South University, No.138, Tongzipo Road, Changsha, 410013 Hunan China; 9 Research Institute of Chinese Medicine, Hunan Academy of Chinese Medicine, No.58, Lushan Road, Changsha, 410000 Hunan China

**Keywords:** Synthesized chrysotile nanomaterials, Gene therapy, Targeted delivery, CircPRMT5, SiRNA, Bladder cancer

## Abstract

**Background:**

Circular RNAs (circRNAs) have important functions in many fields of cancer biology. In particular, we previously reported that the oncogenic circRNA, *circPRMT5*, has a major role in bladder cancer progression. Therapy based on circRNAs have good prospects as anticancer strategies. While anti-circRNAs are emerging as therapeutics, the specific in vivo delivery of anti-circRNAs into cancer cells has not been reported and remains challenging.

**Methods:**

Synthesized chrysotile nanotubes (SCNTs) with a relatively uniform length (~ 200 nm) have been designed to deliver an siRNA against the oncogenic circPRMT5 (si-circPRMT5) inhibit circPRMT5. In addition, the antitumor effects and safety evaluation of SCNTs/si-circPRMT5 was assessed with a series of in vitro and in vivo assays.

**Results:**

The results showed that SCNTs/si-circPRMT5 nanomaterials prolong si-circPRMT5’s half-life in circulation, enhance its specific uptake by tumor cells, and maximize the silencing efficiency of *circPRMT5*. In vitro, SCNTs encapsulating si-circPRMT5 could inhibit bladder cancer cell growth and progression. In vivo, SCNTs/si-circPRMT5 inhibited growth and metastasis in three bladder tumor models (a subcutaneous model, a tail vein injection lung metastatic model, and an in situ model) without obvious toxicities. Mechanistic study showed that SCNTs/si-circPRMT5 regulated the miR-30c/SNAIL1/E-cadherin axis, inhibiting bladder cancer growth and progression.

**Conclusion:**

The results highlight the potential therapeutic utility of SCNTs/si-circPRMT5 to deliver si-circPRMT5 to treat bladder cancer.

**Supplementary Information:**

The online version contains supplementary material available at 10.1186/s40824-022-00251-z.

## Background

One of the most common urinary system malignancy is bladder cancer, which has become increasingly prevalent worldwide [1]. Bladder cancer mortality and morbidity rank top among all urinary system tumors in China [2]. Although advances in surgical treatments and new combination chemotherapy protocols have improved median survival, approximately 50% of patients with bladder cancer develop recurrent/metastatic disease within 2 years of diagnosis [3]. The high probability of recurrence of non-muscle invasive bladder cancer and the poor survival rate of muscle invasive bladder cancer [4, 5] mean that new therapeutic approaches to treat bladder cancer are urgently required.

Recently, among the newly developed cancer treatment methods, gene therapy has emerged as a research hotspot. In particular, next generation sequencing has led to the discovery of circular RNA (circRNAs), which now occupy a central position in research into cancer genomics and gene therapy [6, 7]. CircRNAs are a class of circular non-coding RNA molecules that form a covalently closed continuous loop without free caps and poly(A) tails [8, 9], CircRNAs are involved in the regulation of gene expression and, in cancer, they have demonstrated the potential to regulate progression, metastasis, angiogenesis, apoptosis, and proliferation [10, 11]. Our previous study revealed that *circPRMT5* is highly expressed in bladder cancer tissue and correlates positively with advanced clinical stage and worse survival in patients with bladder cancer [12].

RNA interference (RNAi) is a useful method to suppress RNA/gene expression in a sequence-specific manner and has marked potential in cancer treatment [13–15]. However, in the physiological environment, small interfering RNAs (siRNAs) are degraded rapidly and show poor uptake by cells; therefore, the therapeutic delivery of siRNAs remains a challenge. Currently, there are only 11 clinical formulations comprising RNAi therapeutics [16, 17]. Moreover, similar to other siRNAs, si-circPRMT5 showed a low in vivo transfection efficiency [18]. As far as we know, there have been no reports of the delivery of anti-circRNAs and/or si-circRNAs by nanoparticles into cancer cells. In circRNA therapy, the development of a low toxicity, highly efficient delivery system is a major challenge. Although nonviral vectors represent a valid choice, the agents in current use are limited by their high toxicity and low transfection efficiency [19, 20]. In cancer treatment, a wide variety drug carriers comprising nano-vectors have been reported [21]. In clinical trials, some nano-vectors incorporating drugs demonstrated efficient cancer treatment by virtue of passive nanoparticle accumulation in tumors achieved via their improved retention and permeability, and reduced systemic side effects [22]. Herin, synthetic hydrosilicate chrysotile with the hollow multi-layer nanotube structure, several hundred nanometers in length and a little toxic potential [23], have been incorporated in si-circPRMT5 delivery system for enhance encapsulation efficiency, delivery efficiency and against degradation in circulation et al. Chrysotile, also known as chrysotile asbestos, is widely used in industry. Long-term exposure to asbestos is subject to cause chronic pleural disease, pulmonary fibrosis and lung cancer, which strongly depending on length-diameter ratio and content of metal elements. For example, lung cancer and pulmonary fibrosis could be induced by long-term exposure to asbestos with a length great than 15 μm and a thickness more than 0.1 μm, while mesotheliomas and pleural plaques was usually caused by long-term exposure to asbestos with greater length than ∼4–5 μm and less thickness than ∼0.1 μm. With recent progress in synthetic chrysotile, various synthetic models have been incorporated, such as ion substitution, composition selection, shape control, and internal and external surface characteristic regulation et al. [24]. As reported in literatures, synthetic hydrosilicate chrysotile with several hundred nanometers length have much less toxic than natural chrysotile with over 10 μm length. Therefore, in the present study, we aimed to assess whether the delivery of si-circPRMT5 using geoinspired synthetic chrysotile nanotubes (SCNTs) as a nano-vector has therapeutic efficacy against bladder cancer.

## Methods

### Cell lines, cell culture, and materials

The bladder cancer (T24) cells were purchased from the ATCC (Manassas, VA, USA) and cultured using Dulbecco’s modified Eagle’s medium (DMEM) supplemented with 10% fetal bovine serum (FBS) at 37 °C in a 5% CO_2_ incubator. 4′,6-diamidino-2-phenylindole (DAPI) was acquired from Molecular Probes (Eugene, OR, USA). Dojindo Laboratories (Kumamoto, Japan) supplied the cell counting kit 8 (CCK-8), and BD PharMingen (Heidelberg, Germany) supplied the Annexin V-fluorescein isothiocyanate (FITC) Apoptosis Detection kit. Genepharma (Shanghai, China) synthesized and supplied the siRNAs. The *circPRMT5*-targeting *siRNA* consisted of a sense strand 5′-AUCUUCCGGCUCCUCAAGUUCTT-3′ and an antisense strand 5′-GAACUUGAGGAGCCGGAAGAUTT-3′. The scrambled negative control siRNA (NC)-siRNA also consisted of a sense strand 5′-UUCUCCGAACGUGUCACGUUU-3′ and an antisense strand 5′-ACGUGACACGUUCGGAGAAUU-3′. The sequence of the FAM and Cy5-labeled (fluorescent) NC-siRNAs was the same, and the dye was attached to the antisense strand.

### Preparation of SCNTs-siRNA and characterization

Chrysotile preparation: Using tetraethoxysilane (Si (OCH_3_)_4_) as the silicon source and magnesium chloride hexahydrate (MgCl_2_·6H_2_O) as magnesium source, MgCl_2_·6H_2_O was dissolved in deionized water at the ratio of Mg:Si = 3:2 to form a solution, then Si (OCH_3_)_4_ was added and stirred for 15 min to prepare a 0.1 M solution in 50 mL. The pH of the solution was adjusted to 13.0 by dropping 1 mol/L and 4 mol/L NaOH solution and then stirring for 20 min. The solution was poured into a reactor for hydrothermal synthesis. The synthetic chrysotile sample was obtained by constant temperature reaction at 240 °C for 72 h, repeated filtration and drying in an oven at 60 °C for 6 h.

SiRNA loading: Chrysotile was put into an autoclave for high temperature sterilization, kept at 121 °C for 20 min, cooled to 60–70 °C for sampling. After centrifuging siRNA at 3000 rpm for 5 min, and injecting DEPC water and siRNA into alcohol, the experiment was carried out on the sterile operation platform. Add 62.5 μL of DEPC water into the siRNA NC tube (containing 0.5 OD siRNA), and repeatedly wash with a pipette gun for 2 to 3 times, mix evenly, and place all samples in the same centrifuge tube. Then, 12 μL siRNA and 30 μL chrysotile solution and blank DEPC water were mixed evenly, sealed with sealing film, and then pricked with syringe needle to ensure that the pressure was below 0.8 and vacuum impregnated for 3 h to obtain RNA loaded chrysotile.

### Gel electrophoresis

The ability of SCNTs to bind to the siRNA was assessed using agarose gel electrophoresis. Agarose gels (0.5%, run at 90 V for 60 min) were used to separate 15 μL of SCNTs/siRNA nanoparticles with different mass ratios (1–80), with a control comprising free siRNA. Ethidium bromide staining was used to visualize the nucleic acids on the gel.

### Cellular uptake assay

BD FACSCalibur flow cytometry (BD Biosciences San Jose, CA, USA) was used to quantify the uptake of SCNTs/FAM-si-circPRMT5 nanoparticles by cells. T24 cells (2 × 10^5^ cells/well in 2 mL of DMEM) were added to the wells of six-well plates and grown for 1 d. Phosphate-buffered saline (PBS) was used to rinse the cells, which were then incubated with SCNTs/FAM-si-circPRMT5 nanoparticles for 2 h. Thereafter, cold PBS was used to rinse the cells three times, and then the cells were centrifuged, resuspended, and assessed using flow cytometry.

Next, T24 cells (5 × 10^4^ cells per well) were seeded in chambered coverslips and cultured for 24 h. Subsequently, SCNTs/FAM-si-circPRMT5 nanoparticles at a 20:1 mass ratio was added and incubated for 4 h, after which the culture medium was discarded. The cells were washed twice using PBS and then fixed using 4% paraformaldehyde (v/v). DAPI was used to stain the cell nuclei. Mounting medium was used to seal the cells, which were examined via confocal laser scanning microscopy (CLSM; Nikon, Tokyo, Japan).

### Lysosomal escape

To study the SCNTs/FAM-si-circPRMT5 nanogel’s endosomal escape properties, we seeded 5 × 10^5^ T24 cells (per well) into CLSM dishes and grew them for 1 d at 37 °C. The cells were then incubated with 100 nM SCNTs/FAM-si-circPRMT5 for 2 h. Afterwards, the SCNTs/FAM-si-circPRMT5-containing medium was discarded and fresh medium was added to the cells and incubated for 3 and 6 h, respectively. The cells were then washed with PBS three times and incubated for 30 min with 250 nM LysoTracker Red. The cells were then observed immediately using CLSM.

### Confocal imaging of pathways related to endocytosis

In glass bottom confocal dishes, 5 × 10^4^ T24 cells per well were and incubated overnight. Next day, Alexa Fluor 555-labeled endocytic markers (5 μg/mL Cyclotraxin B (CTX-B), 25 μg/mL transferrin, and 25 μg/mL dextran) and SCNTs/FAM-si-circPRMT5 were added to the cells and incubated for 2 h at 37 °C. During the last 15 min of incubation, Hoechst 33342 at 2 μg/mL was added. At the end of the 2 h incubation, the cells were washed and subjected to confocal microscopy visualization.

### Quantitative real-time reverse transcription PCR (qRT-PCR)

The Trizol reagent was used to extract total RNA from cells or tissues. Total RNA (5 μg) was subjected to reverse transcription using a first-strand cDNA synthesis kit (Beyotime Institute of Biotechnology, Jiangsu China) following the supplier’s protocols. The resultant cDNA was then used as the template for qPCR. The qPCR primers were as follows: circPRMT5 sense: 5′-CCACTGTACTCCTCTGTGTGT-3′ and circPRMT5 anti-sense: 5′- CCACTGTACTCCTCTGTGTGT-3′; miR-30c sense: 5′- ACCATGCTGTAGTGTGTGTAAACA-3′ and miR-30c anti-sense: 5′- TCCATGGCAGAAGGAGTAAA-3′; GAPDH sense: 5′- TGCACCACCAACTGCTTAGC-3′ and GAPDH anti-sense: 5′- GGCATGGACTGTGGTCATGAG-3′.

### Western blotting analysis

Total proteins were obtained from tissues and transfected cells by incubation for 30 min on ice with Radioimmunoprecipitation assay (RIPA) cell lysis buffer containing protease inhibitors, with gentle shaking. After centrifugation of the lysates at 12000 rpm for 10 min, the supernatant was retained and the protein concentration was measured using a bicinchoninic acid (BCA) Protein Assay Kit (Beyotime, Shanghai, China). 8% SDS-PAGE was used to separate the proteins, which were then electrotransferred to polyvinylidene fluoride (PVDF) membranes. The membranes were blocked and then were incubated with primary antibodies at overnight at 4 °C: Anti-SNAIL1 (1:1000 dilution, Proteintech, Chicago, IL, USA), anti-E-cadherin (1:1000 dilution, Abcam, Cambridge, MA, USA), or anti-GAPDH (1:5000 dilutions, Proteintech). Next, the membranes were reacted for 2 h with goat anti-rabbit IgG-horseradish peroxidase (HRP) secondary antibodies. The immunoreactive proteins’ fluorescent signals were assessed using the Odyssey Infrared Imaging System (LI-COR, Lincoln, NE, USA).

### Staining via immunohistochemistry (IHC)

Proteins were stained using primary antibodies and their expression was revealed utilizing a Dako Real Envision Kit (Dako, Carpentaria, CA, USA) following the supplier’s instructions. The staining intensity was scored manually by two experience pathologists independently. To evaluate IHC staining, we used semiquantitative scoring criteria, incorporating the staining intensity and positive areas. A staining index (scores 0–12), obtained as the intensity of SNAIL1, E-cadherin, or Ki67 positive staining (negative = 0, weak = 1, moderate = 2, or strong = 3 scores) and the proportion of immunopositive cells of interest (< 10% = 1, 10–50% = 2, > 50% and < 80% = 3, ≥ 80% = 4), was calculated. In the case of heterogeneous staining, the percentage of different staining intensities was determined individually in each area and the total sum was calculated. The primary antibodies used comprised: Anti-SNAIL1 (1:100 dilution, Proteintech), anti-E-cadherin (1:200 dilution, Abcam), anti-Ki67 (1:30 dilutions, Cell Signaling Technology, Danvers, MA, USA).

### 3-(4,5-dimethylthiazol-2-yl)-2,5-diphenyltetrazolium bromide (MTT) cytotoxicity analysis

We performed MTT assays on T24 cell. Cells at 5000 cells/well were seeded into 96-well plates 24 h prior to the assay. Cells were treated for 48 h, after which they were washed three times with PBS, and 100 μL of fresh medium was added to each well. Next we assed 20 μL of MTT solution (5 mg/mL in deionized water) to the cells and incubated them for 4 h. The medium was discareded, acid SDS solution (100 μL of 10% SDS, 5% isopropanol, 0.012 mol/L HCl) was added to every well and mixed with the purple formazan crystals using transfer-pipettes, and then incubated for 12 h. A microplate reader (Bio-Rad, Hercules, CA, USA) read the optical densities at 570 nm.

### Colony formation assay

In a six-well plate, 1 × 10^4^ cells in 0.4% Seaplague agar were spread on a base of 0.6% agar and grown. Three weeks later, colonies comprising > 80 cells were counted and the results were expressed as the means ± SD of triplicate wells in the same experiment.

### Cell cycle and apoptosis assays

First, T24 cells were grown to 80% confluence in six-well plates, and then treated with PBS, SCNTs, SCNTs/siNC, and SCNTs/si-circPRMT5 for 1 or 2 d. The cells were then assayed following the protocols of the Annexin V-FITC Apoptosis Detection Kit and Cell Cycle and Apoptosis Analysis Kit (Beyotime), followed by flow cytometry.

### Terminal deoxynucleotidyl transferase-mediated (dUTP) nick end labeling (TUNEL) assays

Following the various treatments, the mice were killed humanely. Tumor tissues were surgically removed and fixed using 4% paraformaldehyde. The tissues were sectioned (10 μm thick), stained employing an ApopTag® red in situ apoptosis detection kit (Merck Millipore, Darmstadt, Germany), and viewed using confocal microscopy (Carl Zeiss, Jena, Germany).

### Tumor cell motility in vitro

Migration assay: T24 cells were grown in six-well plates to 80% confluency, and then the cell surface was scratched using a 10 μL pipette tip. After two washes with PBS, the medium was swapped with fresh low serum medium (2% FBS). Images were obtained at 0 h and 48 h under a fluorescent microscope (IX51, Olympus, Tokyo, Japan) and compared to determine the scratch width.

Invasion assay: T24 cells (1 × 10^6^ cells/mL; 200 μL) were added into the top chamber of a 24-well Transwell plate (pore size = 8 μm; Corning Star, Cambridge, MA, USA). Complete medium with 10% FBS (500 μL) was placed in the bottom chamber. The chambers were cultured for 1 and 2 days at 37 °C in 5% CO_2_. The unpenetrated cells were wiped off, and the cells in bottom chamber were fixed using 4% paraformaldehyde, stained with Giemsa, and viewed under a microscope. We counted the membrane-penetrating cells in five randomly selected fields of view.

### In vivo distribution assay

Female BALB/c mice (4 weeks old; 18–22 g in weight), were raised under specific pathogen-free (SPF) conditions with free access to water and food. To investigate the SCNTs nanoparticle biodelivery of si-circPRMT5, we injected Cy5-siRNA and the Cy5 fluorescence into the mice, followed by detection using IVIS.

The injection of 0.1 mL of T24 cells (1 × 10^7^ cells) into BALB/c nude male mice right flanks to establish the xenograft tumor model. When the tumor volume reached 200 to 300 mm^3^, the mice were divided randomly into four groups (*n* = 5) and injected intratumorally with SCNTs/Cy5-si-circPRMT5 complexes, SCNTs, naked Cy5-si-circPRMT5, or PBS. A Bio-Real Quick View 3000 imaging system (Bio-Real Sciences, Salzburg, Austria) was then used to scan the mice at 2 and 8 h after injection, using a 1 s exposure time for each image; images were analyzed using Living Imaging software (Bio-Real Sciences). For the tissue distribution study, tail veins were injected with PBS, naked Cy5-si-circPRMT5, SCNTs, and SCNTs/Cy5-si-circPRMT5 complexes. At 2 h and 8 h after intravenous tail vein injection, the mice were sacrificed. Living Imaging software was utilized for analysis of the excised tissues (tumor, spleen, kidney, lung, liver, and heart).

### Assessment of in vivo antitumor effects using a subcutaneous injection model

1 × 10^6^ T24 cells per mouse were injected subcutaneously into the flank region of each mouse to establish the xenograft tumor model. When the T24 tumors reached 100 mm^3^ on the right flank, the mice were divided randomly into four groups (*n* = 5 per group) and treated with PBS, si-circPRMT5, SCNTs, or SCNTs/si-circPRMT5, separately. In each group, treatments were delivered via intratumoral injection once per week for five weeks. Every four days, the mice were weighed and calipers were used to measure the tumors. The tumor volume calculation was: (length × width^2^ / 2). In addition, the mouse survival rate was recorded. At 24 h after the last injection, the mice were sacrificed, and the tumors were excised and photographed. Levels Ki67, SNAIL1, and E-cadherin proteins were detected using western blotting and IHC. TUNEL staining was carried out to assess apoptosis. An EVOS XL Core microscope was used to observe the stained sections.

### In vivo antitumor effects by tail vein injection

To investigate the antitumor activity of SCNTs/si-circPRMT5 in vivo, a SCID mouse tail vein injection model of lung metastasis was constructed using implanted T24 cells. T24 cells (100 μL of cold PBS containing 2.5 × 10^6^ cells) were injected into tail veins to create tumor-bearing BALB/c nude mice. Four weeks after injection, mice were then divided randomly into the following four groups: (1) PBS control, (2) si-circPRMT5s only, (3) free SCNTs, and (4) SCNTs/si-circPRMT5. The treatments were injected weekly for 5 consecutive weeks through the tail vein. After 5 weeks of treatment, CO_2_ euthanasia was carried out and lung tumor tissues were excised and subjected to histological examination.

### In vivo antitumor effects by intravesical instillation

To determine the antitumor activity of SCNTs/si-circPRMT5 in vivo, an in situ model of bladder cancer was constructed. Ether inhalation was used to anesthetize female SD rats, whose bladders were then infused using 0.2 ml N-methyl-Nitrosurea (MNU) (10 mg/mL; Sigma, St. Louis, MO, USA,) employing a 22-gage angiocatheter once every 14 d for five times. For the avoidance of spontaneous micturition, the catheterized rats were kept under anesthesia for about 45 min [25, 26].

After the successful induction of tumors, 40 rats were divided into 4 groups comprising 10 rats per group. The rats were anesthetized and their bladders were instilled with 500 μL of SCNTs/si-circPRMT5, si-circPRMT5 only, an equivalent dose of free SCNTs, or PBS. For the avoidance of spontaneous micturition, the catheterized rats were kept under anesthesia for about 45 min. These treatments were then delivered once weekly for 5 weeks. At 2 days after termination of therapy, the rats were sacrificed humanely, their bladders were removed, weighed, fixed for 1 d in 4% paraformaldehyde, paraffin-embedded, and examined histopathologically. Transverse sections cut from the midportion of the bladder were subjected to hematoxylin and eosin (H&E) staining.

### Safety evaluation

To investigate SCNTs/si-circPRMT5 toxicity, healthy normal mice received i.v. injections of SCNTs/si-circPRMT5, an equal dose of si-circPRMT5 only, an equal dose of free SCNTs, or PBS (*n* = 5 per group). Twenty-four hours later, blood was collected for routine blood tests. Renal and hepatic damage was assessed according to blood serum levels of aspartate aminotransferase (AST), alanine aminotransferase (ALT), total bilirubin (TBIL), total protein (TP), blood urea nitrogen (BUN), and creatinine (CREA). To assay immunotoxicity, enzyme-linked immunosorbent assay (ELISA) kits (Abcam) were employed to quantify the levels of serum cytokines (interleukin (IL)-1β, IL-6, interferon alpha (IFN-α), and tumor necrosis factor alpha (TNF-α)). The mice were sacrificed, their organs (heart, liver, spleen lung, and kidney) were removed, sectioned, and subjected to H&E staining.

### Statistical analysis

GraphPad Prism 5 software (GraphPad Inc., La Jolla, CA, USA) was employed to carry out the statistical analysis. The mean ± SEM or the mean ± SD were utilized to present the numerical data. One-way ANOVA and Tukey’s test together were utilized for the statistical analyses and statistical significance was indicated by a *P*-value < 0.05.

## Results

### Synthesis and characterization of SCNTs

**Fig. 1:**
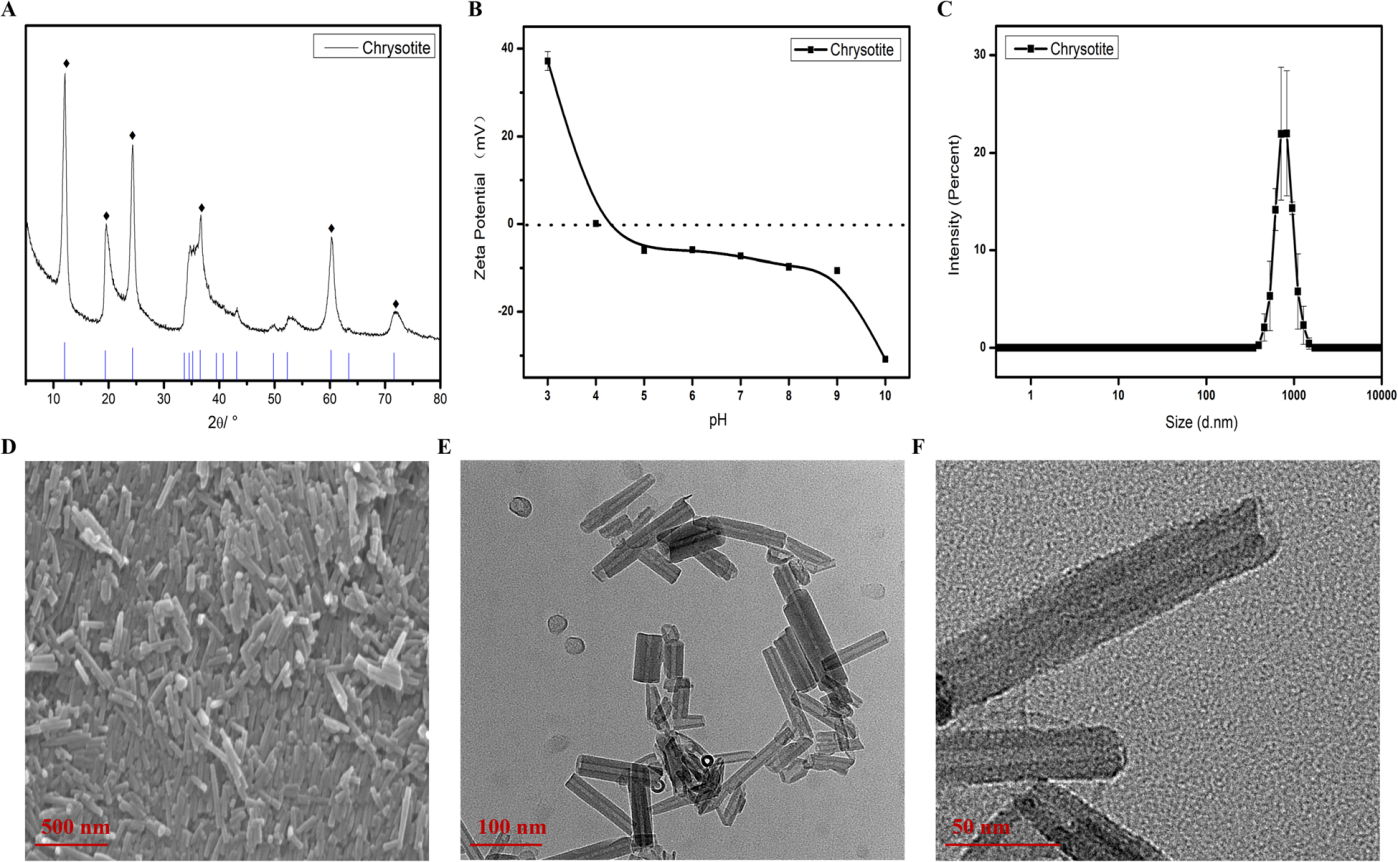
Characterization of the SCNTs. (**A)** XRD patterns, **(B)** Zeta-potential, **(C)** DLS analysis, **(D)** SEM images, and **(E&F)** TEM images of SCNTs

XRD, Zeta potential, DLS analysis, SEM, and TEM of chrysotile has been shown in Fig. 1. It can be seen that the characteristic peak of chrysotile sample in the figure is 12.18°、19.55°、24.33°、36.62°、60.33°, which corresponds to the characteristic peak 12.15°、19.38°、24.35°、36.55°、60.26° in chrysotile standard PDF card one by one, and there is basically no impurity peak, which indicates that the chrysotile reaction obtained at 240 °C for 72 h is completed (Fig. 1A). Zeta potential chart (Fig. 1B) shows that chrysotite has overall electronegativity, and the overall stability increases at pH = 10. The isoelectric point was approximately 4.3, and most of the zeta potentials were negative between 41.0 mV (pH 2.0) and − 24.8 mV (pH 10.0). Zeta potential of chrysotite is close to 0, which indicates that the formation of chrysotite nanotubes is not caused by electrostatic action, but by chemical combination. And further, the presented zeta potential numerical was the sum of the zeta potential from external surface and internal surface of chrysotile. Synthetic chrysotile nanotube is composed of brucite (Mg (OH)_2_) and tridymite (SiO_2_) layers. The brucite octahedral sheet forms the outer side of the tube and tridymite SiO_2_ groups are anchored to the inner side of the tube. The mixed-charge nanoparticle was covered with positively charged external surface and negatively charged internal surface. The negatively charged siRNA could be effectively anchored on the positively charged external surface through electrostatic interaction, but it was hard to package into the negatively charged internal surface due to the surface tension and the like charges repel. siRNA was loaded into the lumen of synthetic chrysotile nanotubes by vacuum impregnation, subsequent repeating rinsing was performed to remove the outside surface residual. After ultrasonic bubble removal and improved dispersion, one thousandth of sample dilution solution was tested by DLS (Fig. 1C). SEM image (Fig. 1D) showed that the synthesized chrysotile nanotubes have good crystallinity in a wide range. Moreover, the freeze-dried samples are uniform and orderly. TEM images (Figs. 1E and F) show that the synthesized chrysotile nanotubes have perfect crystallization, clear and complete outline, good dispersion, and uniform diameter. The length of the nanotubes is 100–200 nm, a few of them are more than 500 nm, the outer diameter is about 25 nm, and the inner diameter is about 10 nm. At the beginning of the reaction process, the raw material particles will agglomerate and break up, and then begin to dissolve due to the high free energy. At the same time, these particles are equivalent to crystal seeds, and the dissolved particles are deposited again, and the crystals begin to grow at the agglomerations. With the extension of reaction time, the surface energy tends to decrease due to the different solubility of large particles and small particles, resulting in the continuous growth and integrity of large grains and the gradual disappearance of smaller grains. With the extension of reaction time, the crystallinity of crystals becomes higher.

### SCNTs siRNA-binding efficiency

**Fig. 2:**
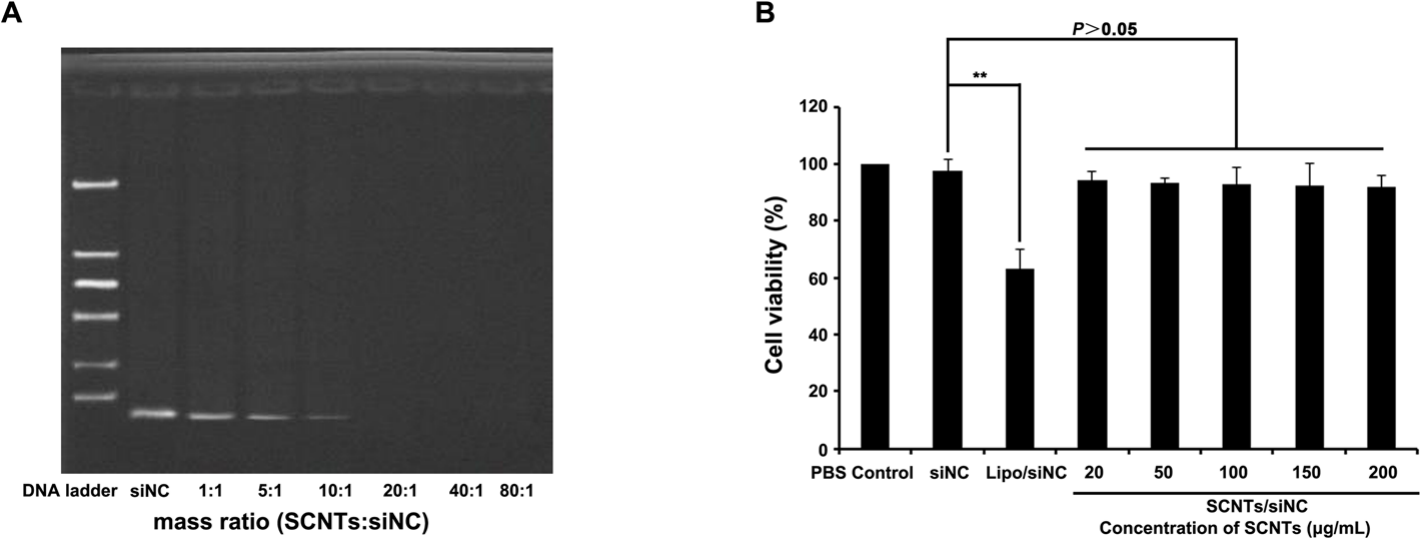
Formulation of SCNTs/siRNA and assessment of non-specific cytotoxicity. (**A)** Gel retardation results of different weight ratios of SCNTs loaded with siRNA. (**B)** SCNTs/siRNA cytotoxicity assays. SCNTs/siRNA at different SCNT concentrations were incubated with cells for 48 h. A CCK-8 kit was used to assess cell viability. Data are presented as mean ± SD from three independent experiments, which were performed in triplicate. **P < 0.01

As the mass ratio of SCNTs and siRNA increased, gel retardation assays showed that the amount of unbound siRNA decreased (Fig. 2A). At a mass ratio of 20, the unbound siRNA band disappeared, which suggested that at that mass ratio, these nanoparticles can bind siRNA efficiently. Therefore, nanoparticles at a mass ratio of 20 were used in further in vitro and in vivo experiments. The experimental group was treated with SCNTs/si-circPRMT5 or SCNTs/FAM-si-circPRMT5, the control groups were treated with PBS, SCNTs, SCNTs/siNC, si-circPRMT5 or FAM-si-circPRMT5.

### Evaluation of toxicity of SCNTs

We investigated the toxicity of SCNTs nanoparticles at different concentrations (20, 50, 100, 150, and 200 μg/mL) and found that cell viability was over 85% for SCNTs nanoparticles even at the highest concentration (200 μg/mL), which was not caused by the toxicity of the SCNTs (Fig. 2B). In contrast, at the transfection effective dose, Lipofectamine 3000 demonstrated some cytotoxicity. Note that SCNTs concentrations above 50 μg/mL were not used during the in vivo studies and were tested only to demonstrate that the synthesized SCNTs were nontoxic even at the highest concentrations.

### SCNTs transfection efficiency

**Fig. 3:**
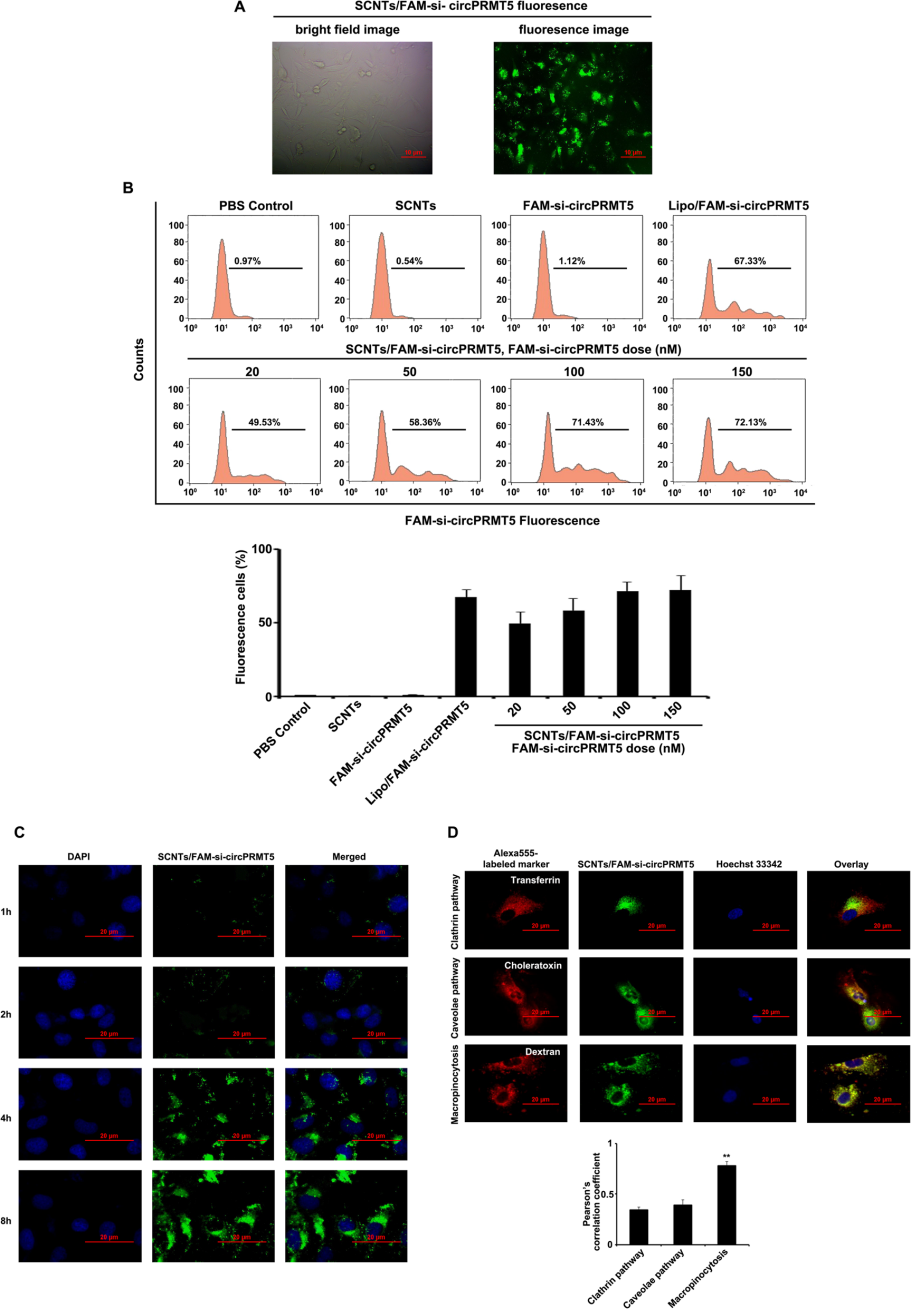
Uptake and distribution of SCNTs/FAM-si-circPRMT5 by bladder cancer cells. (**A)** Images of SCNTs/FAM-si-circPRMT5 fluorescence at 6 h post-transfection acquired using fluorescence and bright field microscopy. (**B)** Cell uptake of SCNTs/FAM-si-circPRMT5 with different siRNA contents by flow cytometry. Representative histograms are shown in the upper panel, the lower panel shows the mean ± SD (*n* = three independent experiments). (**C)** Confocal laser scanning microscopy (CLSM) images of the uptake of SCNTs/FAM-si-circPRMT5 by T24 cells at 1, 2, 4, and 8 h. FAM, green signal; DAPI (4′,6-diamidino-2-phenylindole), blue signal; Scale bar = 25 μm. (**D)** Representative confocal microscopy image showing the co-localization of SCNTs/FAM-si-circPRMT5 (green) with endocytic markers (dextran, cholera toxin, and transferrin) labeled with Alexa Fluor 555 (red). Hoechst 33342 (blue) was used to counterstain the nuclei (upper panel). Pearson’s correlation coefficient analysis of The co-localization between endocytosis markers and SCNTs/FAM-si-circPRMT5 in T24 cells using Pearson’s correlation coefficient analysis in Image J Coloc2 (lower panel). **P < 0.01

Inverted fluorescence microscopy was used to determine T24 cell uptake of SCNTs/fluorescein (FAM)-si-circPRMT5 complexes. FAM-si-circPRMT5 were observed inside T24 cells after 4 h of incubation, showing internalization of SCNTs (Fig. 3A).

To ascertain the influence of the composition of the formulations on si-circPRMT5 internalization, T24 cells were incubated for 4 h with various concentrations of SCNTs/FAM-si-circPRMT5, followed by flow cytometry after the extracellular fluorescence was quenched. We observed an SCNTs/FAM-si-circPRMT5 dose-dependent increase in the proportion of fluorescent cells (i.e., those containing SCNTs/FAM-si-circPRMT5 complexes) (Fig. 3B). Moreover, the transfection efficiency using SCNTs/FAM-si-circPRMT5 at 100 nM was similar to that gained using Lipofectamine 3000/FAM-si-circPRMT5 complexes. At concentrations of si-circPRMT5 up to 150 nM, little change in the efficiency of transfection was observed. Thus, the optimal dose of SCNTs/FAM-si-circPRMT5 to transfect T24 cells was 100 nM, which showed a similar transfection efficiency to that of Lipofectamine 3000.

### Uptake by bladder cancer cells, intracellular trafficking, and escape from endosomes of SCNTs/FAM -si-circPRMT5

Uptake by cells is associated closely with the siRNA delivery efficiency [27, 28]. Generally, high uptake of siRNA by cells leads to high efficiency of gene silencing. Herein, the cellular uptake of SCNTs/FAM -si-circPRMT5 in T24 cells was observed using a fluorescence microscope. FAM-siRNA-derived green fluorescence was observed in T24 cells exposed to SCNTs/FAM-si-circPRMT5 for 1 h, indicating that the T24 cell could internalize the nanoparticles, regardless of the active targeting moieties (Fig. 3C). With the prolongation of incubation to 4 and 8 h, the fluorescence intensity in the SCNTs/FAM-si-circPRMT5-treatment group increased.

To determine whether the T24 took up the SCNTs-siRNA through endocytosis, T24 cells were incubated with SCNTs/FAM-siRNA in the presence of Alexa Fluor 555-labeled endocytic markers, followed by by confocal microscopy analysis. Image J with Pearson’s correlation coefficient analysis was used to quantify the colocalization of endocytic markers and the SCNTs/FAM-siRNA. In T24 cells, marked co-localization of Dextran (a marker of macropinocytosis) with SCNTs/FAM-siRNA was observed (Fig. 3D), with a Pearson’s correlation coefficient of approximately 0.8 (Fig. 3D). By contrast, we observed significantly less co-localization of SCNTs/FAM-siRNA with cholera toxin (a caveolae-mediated endocytosis marker) or transferrin (a clathrin-mediated endocytosis marker) (Fig. 3D), indicating that T24 cells mainly took up SCNTs/FAM-siRNA via macropinocytosis.

Next, T24 cells were stained with LysoTracker and observed using to confocal fluorescence microscopy to determine the escape of SCNTs/si-circPRMT5 from lysosomes (Fig. S1). In the transfected cells, SCNTs/si-circPRMT5 (green) and LysoTracker (red) fluorescence were co-localized after 1 h (Fig. S1), demonstrating the SCNTs/si-circPRMT5 were located in the lysosomes. After 4 h of incubation, the red (LysoTracker) and green (SCNTs/si-circPRMT5) fluorescence had separated (Fig. S1), which demonstrated that the SCNTs/si-circPRMT5 has escaped from the lysosomes into the cytoplasm.

### circPRMT5 knockdown in bladder cancer cell reduces cell proliferation and colony formation

**Fig. 4:**
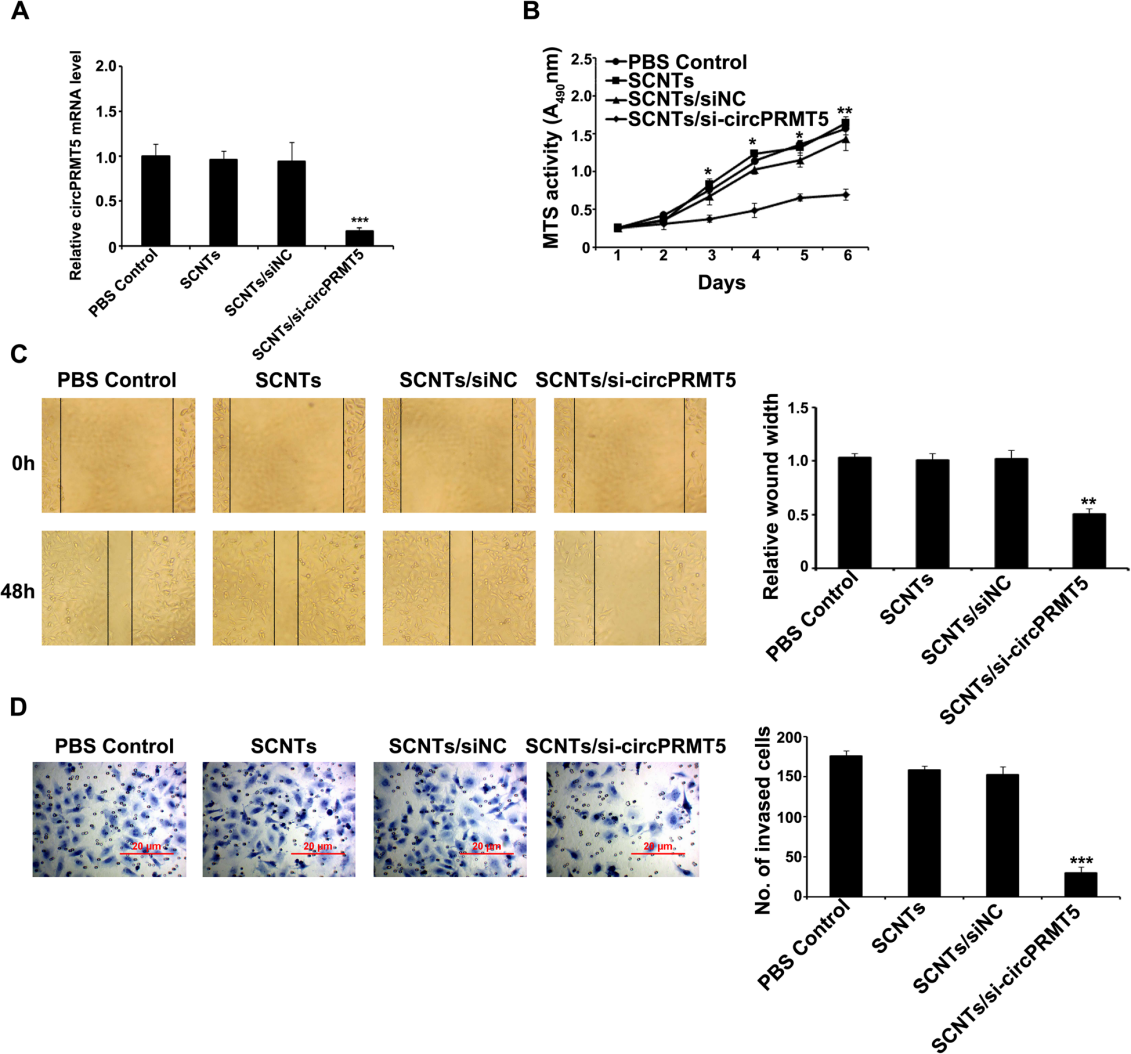
circPRMT5 knockdown by SCNTs/si-circPRMT5 reduces the growth, invasion and migration and invasion capacity of bladder cancer cells in vitro. (**A)** Semi-quantitative RT-PCR was used to assess *circPRMT5* expression in the SCNTs/si-circPRMT5 and other treatment groups. (**B)** Effects of *circPRMT5* silencing by SCNTs/si-circPRMT5 on T24 bladder cancer cells proliferation, detected by CCK-8 assay. (**C)** Wound-healing assays to measure T24 bladder cancer cell migration (photographed at 0 and 48 h post-wounding). (**D)** Transwell assays T24 bladder cancer cell invasion; photographs were taken at 48 h after the cells were incubated in a Matrigel pre-coated Transwell chamber. *P < 0.05, **P < 0.01, ***P < 0.001

Cancer is characterized by dysregulated cell proliferation and enhanced colony formation [29, 30]; therefore, the effect of *circPRMT5* silencing on T24 cell growth was assessed using a CCK-8 assay. The results showed effective suppression of T24 cell growth by SCNTs/si-circPRMT5 at the RNA level (Fig. 4A). The proliferation of T24 cells decreased gradually when treated with SCNTs/si-circPRMT5 compared with treatment with PBS, SCNTs, and SCNTs/siNC (Fig. 4B). Next, in the same treatment groups, we assessed the cells’ colony formation capacity. The SCNTs/si-circPRMT5-treated T24 cell types had the lowest colony formation ability among the groups (Fig. S2).

### Bladder cancer cell migration and invasion is regulated by circPRMT5

Previously, we reported a significant association of *circPRMT5* with bladder cancer migration and invasiveness [12]. In a wound healing assay, T24 cells lines transfected with SCNTs/si-circPRMT5 showed significant time-dependent reductions in cell migration, whereas in the scrambled SCNTs control group, no reduction was observed (Fig. 4C). Cells in the SCNTs/si-circPRMT5 group showed decreased invasiveness compared with those in the control groups (Fig. 4D). Thus, *circPRMT5* silencing via SCNTs/si-circPRMT5 inhibits the invasion and migration of bladder cancer cells.

### Silencing of circPRMT5 causes bladder cancer cell apoptosis and S phase cell cycle arrest

To determine whether apoptosis is responsible for *circPRMT5* silencing-induced reduced proliferation of T24 cells, annexin V and PI staining followed by flow cytometry were used to analyze the cells. At 2 d after transfection, in SCNTs/si-circPRMT5-treated cells, the apoptosis rate (annexin-V +/PI - and annexin-V +/PI +) was increased compared with the rate in the controls. Silencing of *circPRMT5* via SCNTs/si-circPRMT5 increased the T24 cell apoptotic percentage by 43.35%, compared with that in the scrambled SCNTs and blank control cells (Fig. S3).

Next, flow cytometry was used to examine the cell cycle of T24 cell lines transfected with SCNTs/si-circPRMT5 particles. Compared with that in scrambled SCNTs controls, the proportion of SCNTs/si-circPRMT5-treated T24 cells in the G1- and S-phases was decreased at 48 h post-transfection. The number of T24 cells treated with SCNTs/si-circPRMT5 in the sub-G1 phase was higher than that in the three control groups (Fig. S4). The Proliferation Index (PI) scores for the PBS, SCNTs, SCNTs/siNC-treated, and SCNTs/si-circPRMT5-treated cells were 0.5289, 0.4799, 0.5017, and 0.3698, respectively (Fig. S4). Overall, our findings indicated that *circPRMT5* silencing via SCNTs/si-circPRMT5 inhibited cancer cell proliferation in vitro via cell cycle arrest and apoptosis induction.

### Biodistribution of SCNTs/FAM-si-circPRMT5 nanocomplexes in vivo via intratumoral injection

**Fig. 5:**
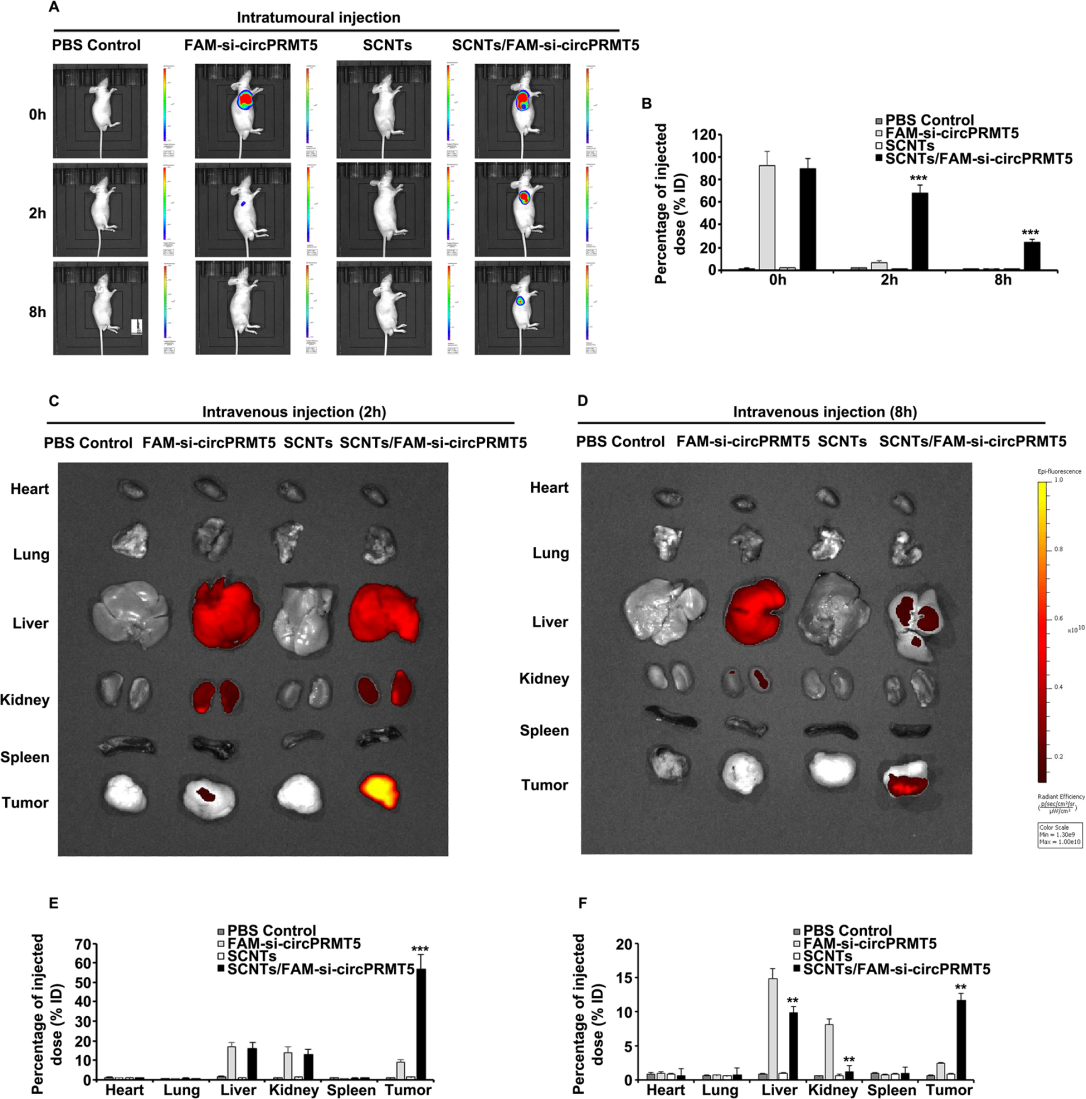
In vivo imaging assessment of the biodistribution of SCNTs/FAM-si-circPRMT5. (**A)** In vivo images of mice bearing T24 tumors treated with SCNTs/FAM-si-circPRMT5 and other complexes after intratumoral injection at 2 h and 8 h. (**B)** Tumor site in vivo fluorescence intensity quantification after intratumoral injection at 2 h and 8 h. Data represent the mean ± SD (*n* = 5). (**C-D)** In vivo image of Cy5 accumulation in tumor and organs after intravenous injection of SCNTs/FAM-si-circPRMT5 and other complexes for 2 h **(C)** and 8 h **(D)**. (**E-F)** Ex vivo fluorescence intensity quantification of control organs and tumors after intravenous injection at 2 h and 8 h. Data show represent mean ± SD (*n* = 5). **P < 0.01, ***P < 0.001

The excellent in vitro results prompted us to hypothesize that SCNTs copolymers have good potential to deliver siRNAs in vivo to treat tumors. The final therapeutic efficacy is markedly affected by the selective enrichment of siRNA complexes within tumors. Therefore, we studied the SCNTs/FAM-si-circPRMT5 nanocomplex biodistribution by intratumoral injection into BALB/c nude mice bearing T24 xenograft tumors. At 2 h after injection, the fluorescence signals from tumors in the SCNTs/FAM-si-circPRMT5 group had increased significantly relative to those of free FAM-si-circPRMT5 and the other groups (Fig. 5A). At 8 h after injection, the SCNTs/FAM-si-circPRMT5 group still displayed tumor fluorescence, whereas we could not see any fluorescence accumulation from free FAM-si-circPRMT5 in the tumor site (Fig. 5A). The percentage of the injected dose of SCNTs/FAM-si-circPRMT5 and free FAM-si-circPRMT5 in the tumor and other major viscera were analyzed at 2 and 8 h quantitatively. Consistently, at both time points, the tumor accumulation of SCNTs/FAM-si-circPRMT5 was significantly higher than that of free FAM-si-circPRMT5 (Fig. 5B). The high fluorescence in the tumor demonstrated that SCNTs/FAM-si-circPRMT5 accumulated mainly in the tumor sites.

### Biodistribution of SCNTs/FAM-si-circPRMT5 nanocomplex in vivo via tail vein injection

Next, we investigated whether the SCNTs could protect the siRNA efficiently in blood circulation, and if they showed an enhanced permeation and retention effect in the tumor. We also wished to verify the feasibility of using SCNTs nanocomplexes to treat tumors in vivo. Therefore, SCNTs/FAM-si-circPRMT5 biodistribution was assessed in T24 tumor bearing mice (*n* = 5) after mouse tail vein injection. An NIR imaging system was used to visualize the in vivo biodistribution. At 2 h and 8 h after intravenous injection, the FAM fluorescence intensity in bladder tumor tissues from the SCNTs/FAM-si-circPRMT5 group was significantly higher than in the free FAM-si-circPRMT5 group (Figs. 5C and D). Moreover, at 8 h post-injection, the liver and kidney FAM fluorescence intensities were greatly decreased in the SCNTs/FAM-si-circPRMT5 compared with those in the FAM-si-circPRMT5 group (Fig. 5D). Interestingly, in both groups, barely detectable fluorescence signals were seen in the lungs, spleen and heart (Figs. 5C and D). We also quantified the percentage of the injected dose of SCNTs/FAM-si-circPRMT5 and free FAM-si-circPRMT5 in tumors and major organs at 2 h and 8 h. At both time points, significantly more SCNTs/FAM-si-circPRMT5 accumulated in the tumor than did FAM-si-circPRMT5, while the liver and kidney clearance of FAM-si-circPRMT5 at 8 h was markedly faster than that of SCNTs/FAM-si-circPRMT5 (Figs. 5E and F).

### In vivo anticancer activity in the subcutaneous model

**Fig. 6:**
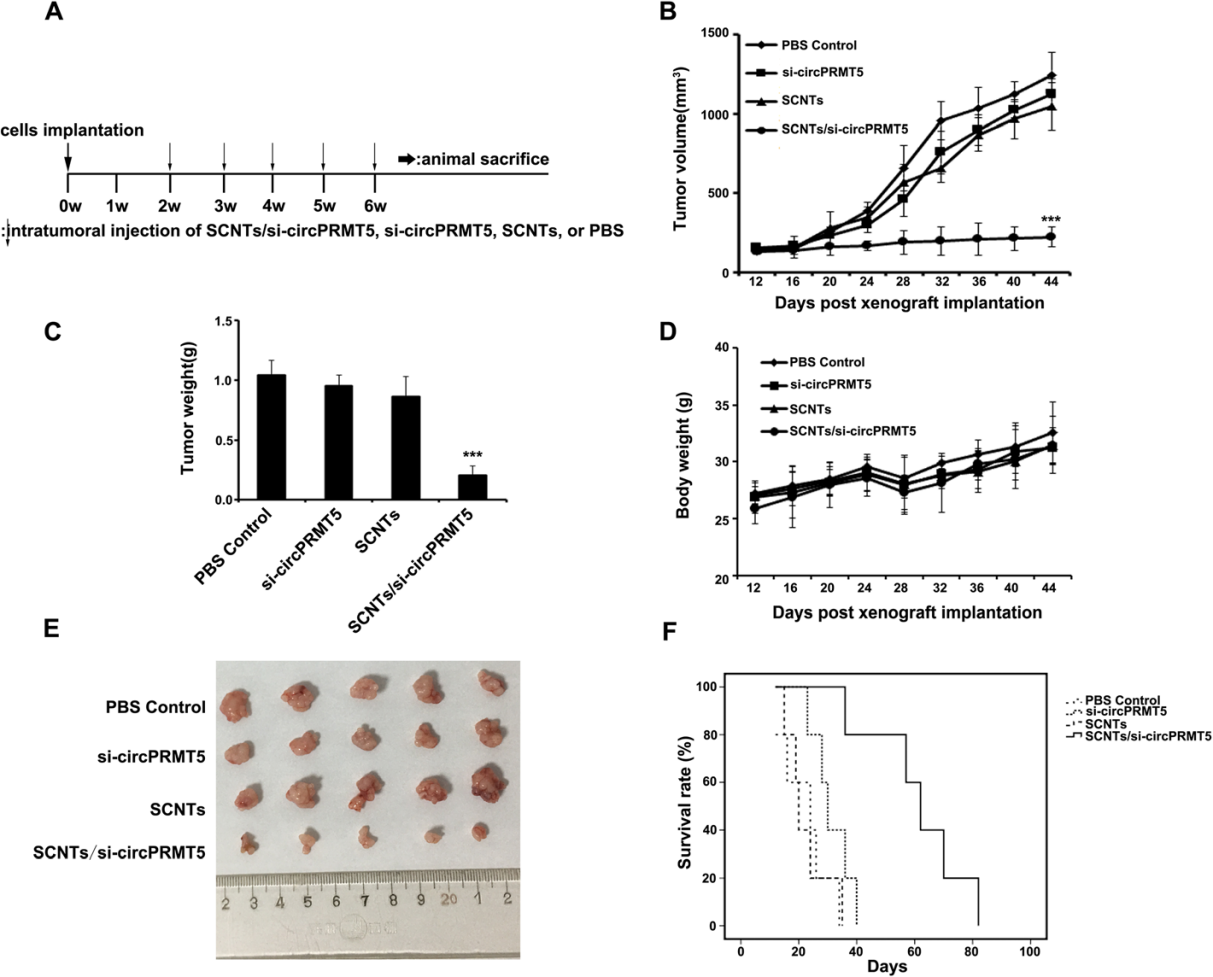
Tumor growth inhibition effects of SCNTs/si-circPRMT5 in the subcutaneous model. (**A)** T24 tumor treatment schedule. (**B)** Growth curves of tumors of mice treated with different therapeutic regimens (*n* = 5). (**C)** The excised tumor tissue weight from all the groups. Data represent the mean ± SD (*n* = 5). (**D)** Body weight changes during treatment. Data represent the mean ± SD (*n* = 5). (**E)** Mouse tumor morphology. (**F)** Survival rate of BALB/c nude mice bearing T24 tumors. ***P < 0.001

The anti-tumor activity of SCNTs/si-circPRMT5 polyplexes was evaluated using a subcutaneous mouse model. T24 cells were injected into the mammary fat pad of female Balb/C mice subcutaneously and allowed to form palpable tumors. When the tumor size reached 100 mm^3^, the tumor-bearing mice were randomized into four groups (*n* = 5 per group) and administered intratumorally with (i) PBS, (ii) si-circPRMT5, (iii) SCNTs, and (iv) SCNTs/si-circPRMT5, following the timeline schedule displayed in Fig. 6A. SCNTs/si-circPRMT5 caused minimal toxicity, as indicted by the lack of significant body weight loss observed during treatment (Fig. 6D). After five dose treatments, tumor growth was retarded dramatically in the SCNTs/si-circPRMT5 group (Figs. 6B, C, and E). Compared with those in the PBS group, mice receiving SCNTs/si-circPRMT5 displayed an 82.24% decrease in tumor size, and an 80.44% decrease in tumor weight (Figs. 6B and C). Contrastingly, neither the SCNTs nor the free si-circPRMT5 groups showed a significant difference in tumor weight or size compared with those in the PBS group (Figs. 6B and C). Moreover, the survival of the mice was prolonged significantly in the SCNTs/si-circPRMT5 group (Fig. 6F).

Mice receiving SCNTs/si-circPRMT5 showed the most appreciable downregulation of *circPRMT5* expression following treatment, as evidenced from homogenized tumor tissues (Fig. S5). IHC of tumor sections for Ki67 revealed markedly reduced Ki67 immunoreactivity in SCNTs/si-circPRMT5-treated tumors compared with that in the other treatment groups (Fig. S6). Furthermore, TUNEL staining revealed an increased abundance of apoptotic cells in the SCNTs/si-circPRMT5 group compared with that in the control groups (Fig. S6). These results support SCNTs as an effective and long-circulating vector to silence *circPRMT5* in vivo.

### In vivo anticancer activity in the tail vein injection model of lung metastasis

**Fig. 7:**
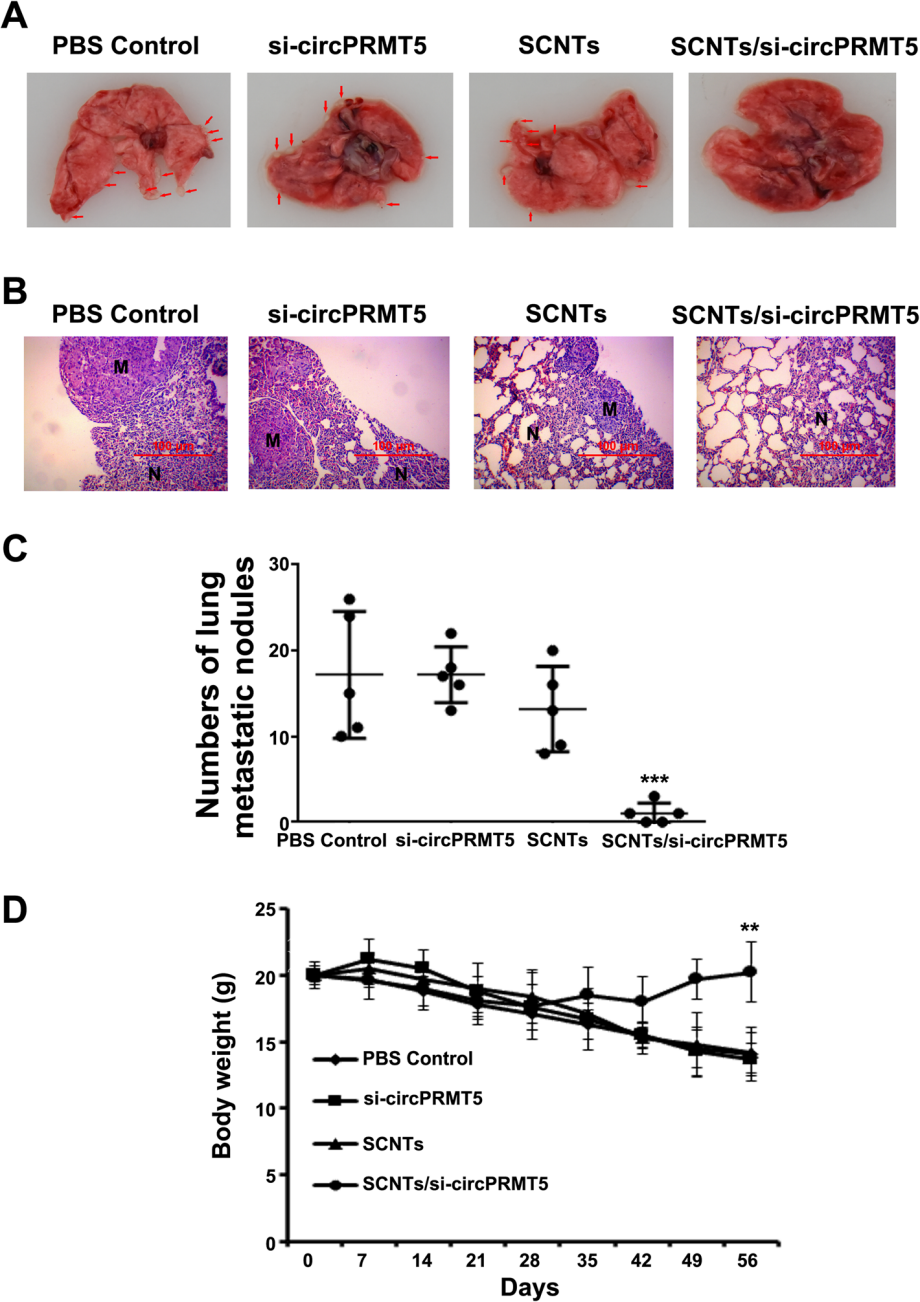
Activity of SCNTs/si-circPRMT5 against metastatic bladder cancer in a mouse model of bladder cancer metastasis constructed via tail vein injection. (**A)** Representative images of T24 tumor-bearing mouse lungs under various treatments. (**B)** Representative H&E-staining of lung sections of mice bearing T24 tumors under various treatments. (**C)** Comparison of T24 tumor-bearing mice pulmonary metastatic nodules under various treatments. (**D)** Changes in body weights of T24-bearing mice treated with various treatments. Data shows represent mean ± SD (*n* = 5). **P < 0.01, ***P < 0.001

To assess the efficacy of SCNTs/si-circPRMT5 therapy in vivo, we used si-circPRMT5 therapeutically in a tail vein injection model of lung metastasis. After the last treatment, the lungs were excised from the mice and examined. Clear signs of lung metastasis were observed in the mice treated with SCNTs, free si-circPRMT5, and PBS, whereas no visible metastasis was detected in the mice treated with SCNTs/si-circPRMT5 (Figs. 7A and B**)**. Notably, in the mice treated with SCNTs/si-circPRMT5, no body weight loss was recorded (Fig. 7D), indicating that SCNTs/si-circPRMT5 caused no adverse effects during their effective inhibition of tumor metastasis. Contrastingly, in the mice treated with PBS, si-circPRMT5, and SCNTs, significant loss of body weight was recorded, which was probably the result of lung malfunctions resulting from metastasis (Fig. 7D). The quantification of pulmonary metastatic nodules further supported the observation that SCNTs/si-circPRMT5 effectively suppressed lung metastasis (Fig. 7C). These results highlighted that SCNTs/si-circPRMT5 is highly efficient, and that targeted delivery of siRNA to metastatic lung tumors had a substantial antimetastatic effect in mice.

### In vivo anticancer activity in the in situ model of bladder cancer

**Fig. 8:**
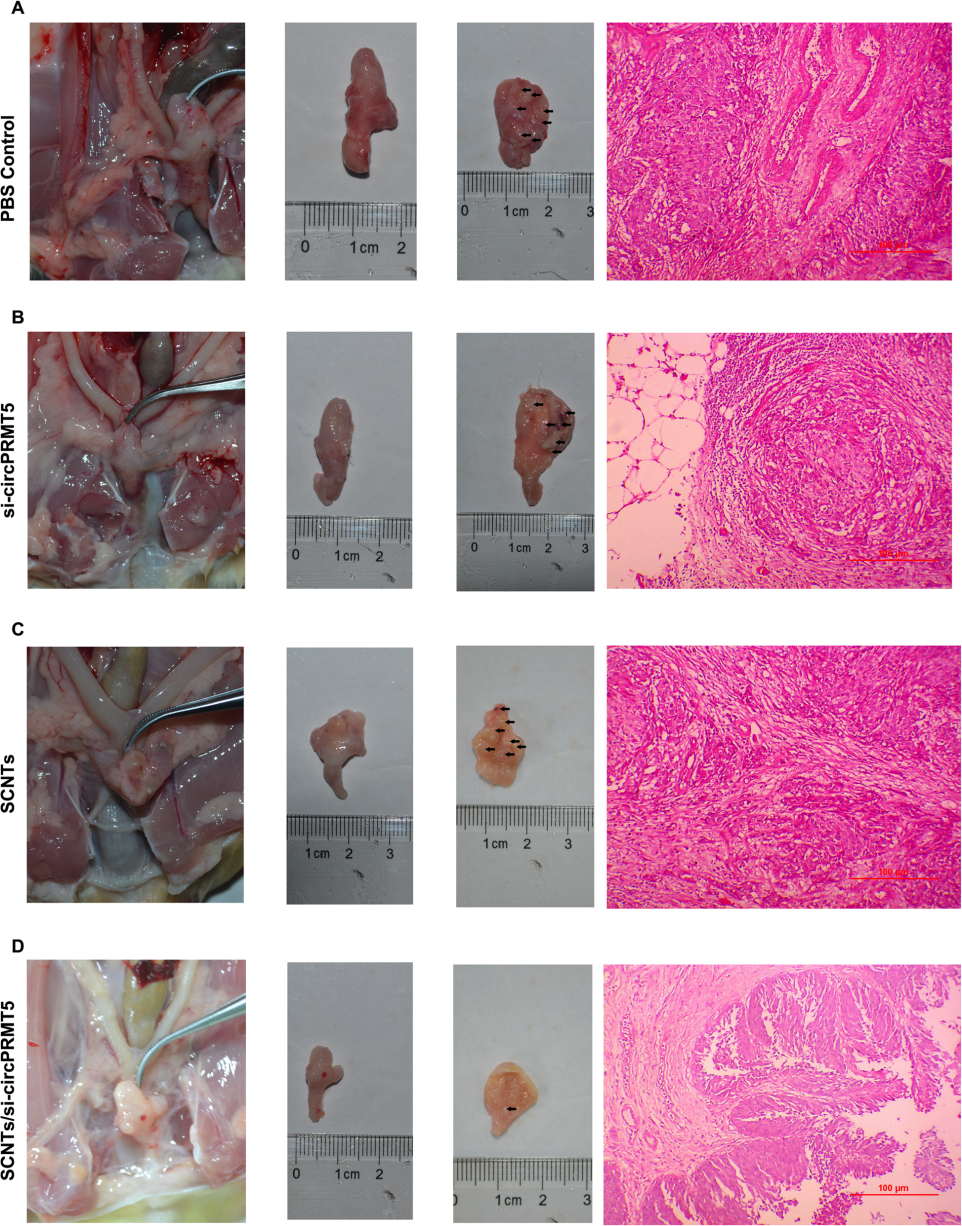
Antitumor effects of SCNTs/si-circPRMT5 in a mouse in situ model of bladder. (**A)** Images of tissue slices stained with H&E and excised bladders (muscle invasive bladder cancer: ≥ stage pT2) in the PBS-treated group. (**B)** Images of tissue slices stained with H&E and excised bladders (muscle invasive bladder cancer: ≥ stage pT2) in the si-circPRMT5 treatment group. (**C)** Images of tissue slices stained with H&E and excised bladders (muscle invasive bladder cancer: ≥ stage pT2) in the SCNT treatment group. (**D)** Images of tissue slices stained with H&E and excised bladders (noninvasive papillary carcinoma: stage pTa) in the SCNTs/si-circPRMT5 treatment group

Urinary bladder instillation chemotherapy is a frequently used therapy to treat bladder cancer. Therefore, we examined the clinical significance of the SCNTs/si-circPRMT5 nanoparticles by assessing their ability to suppress tumors in an in situ bladder cancer model. Histopathology and bladder weight determination showed significant differences among the groups treated with SCNTs/si-circPRMT5, SCNTs, and free si-circPRMT5 (*P* < 0.05) (Table S1). Moreover, SCNTs/si-circPRMT5 treatment resulted in most of the bladder cancers examined having a lower tumor stage (stage pTa/T1), whereas, in the control group, most of the tumors were staged at higher than pT2. This suggested a markedly better therapeutic effect of SCNTs/si-circPRMT5 than PBS treatment. This conclusion was confirmed using H&E staining of tissue slices and histological examination of excised bladders (Fig. 8).

### Evaluating the safety of SCNTs/si-circPRMT5

**Fig. 9:**
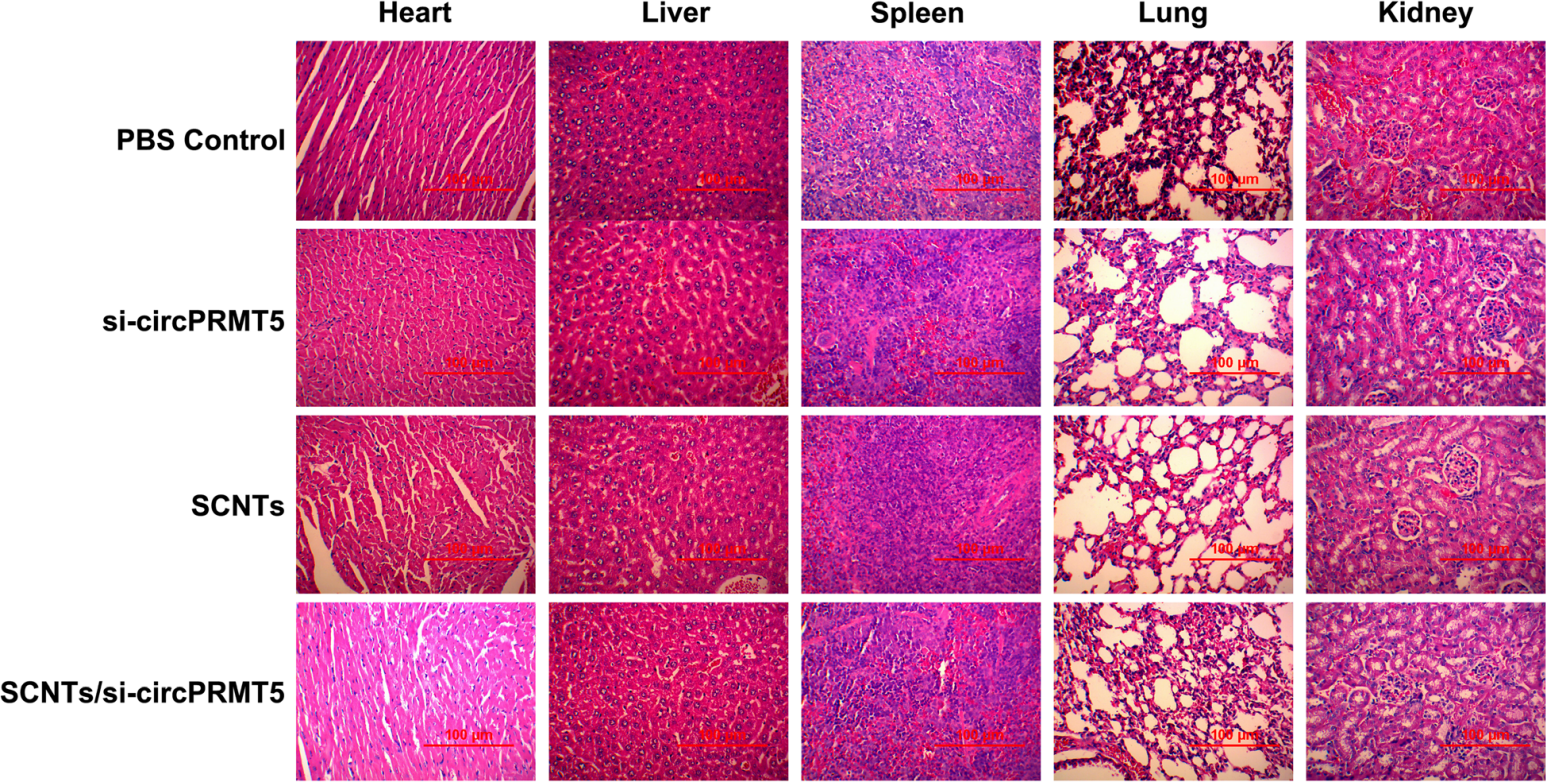
Hematoxylin and eosin-staining of major organs (kidney, lung, spleen, liver, and heart) from mice subjected to SCNTs/si-circPRMT5 treatment and other groups

**Fig. 10:**
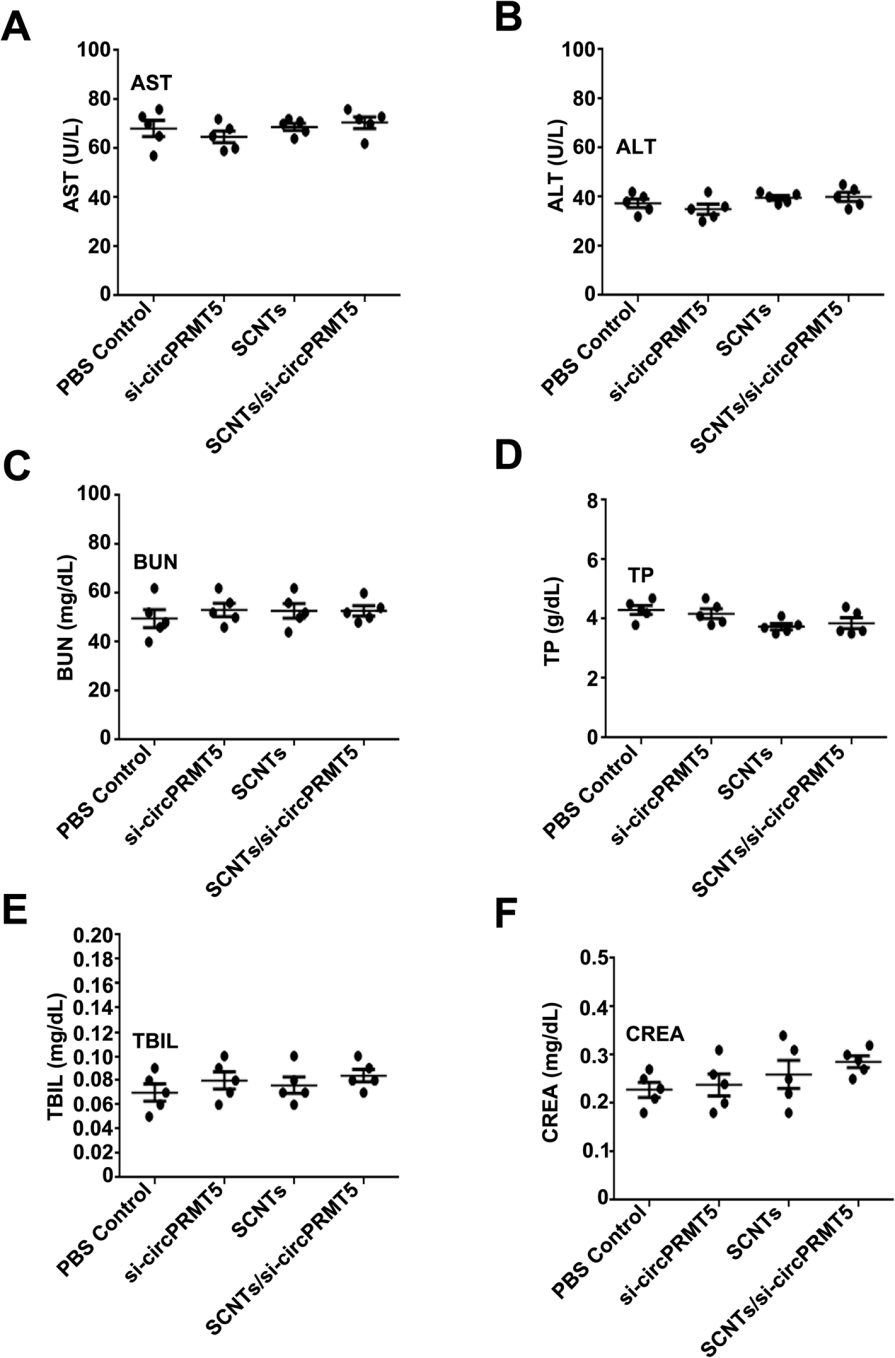
Serum levels of AST (**A**), ALT (**B**), TP (**C**), TBIL (**D**), CREA (**E**), and BUN (**F**) at 1 day after intravenous injection into mice (*n* = 5)

**Fig. 11:**
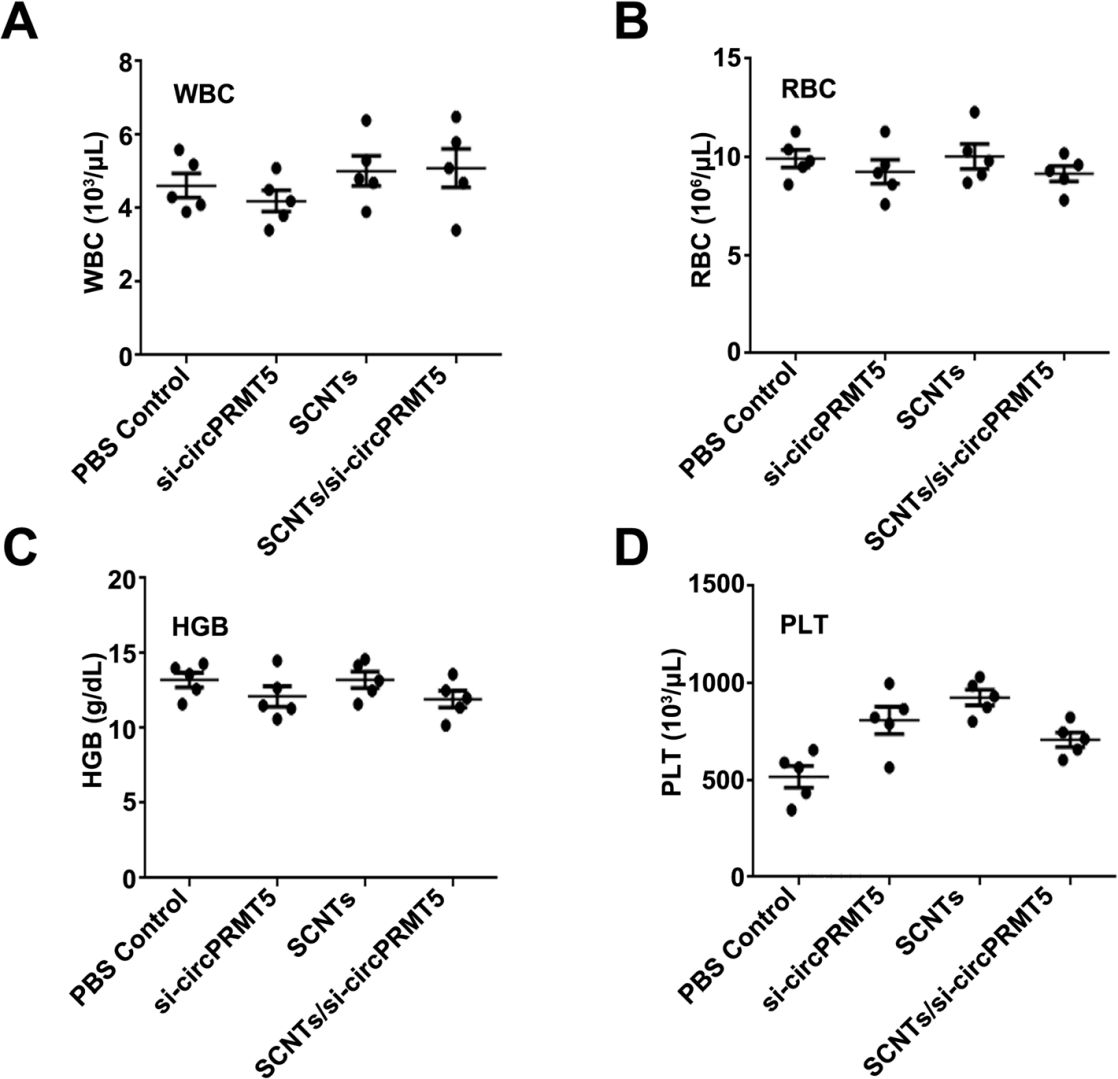
Hematological parameters of WBC(**A**), RBC (**B**), HGB (**C**), and PLT (**D**) at 1 day after intravenous injection into mice (*n* = 5)

**Fig. 12:**
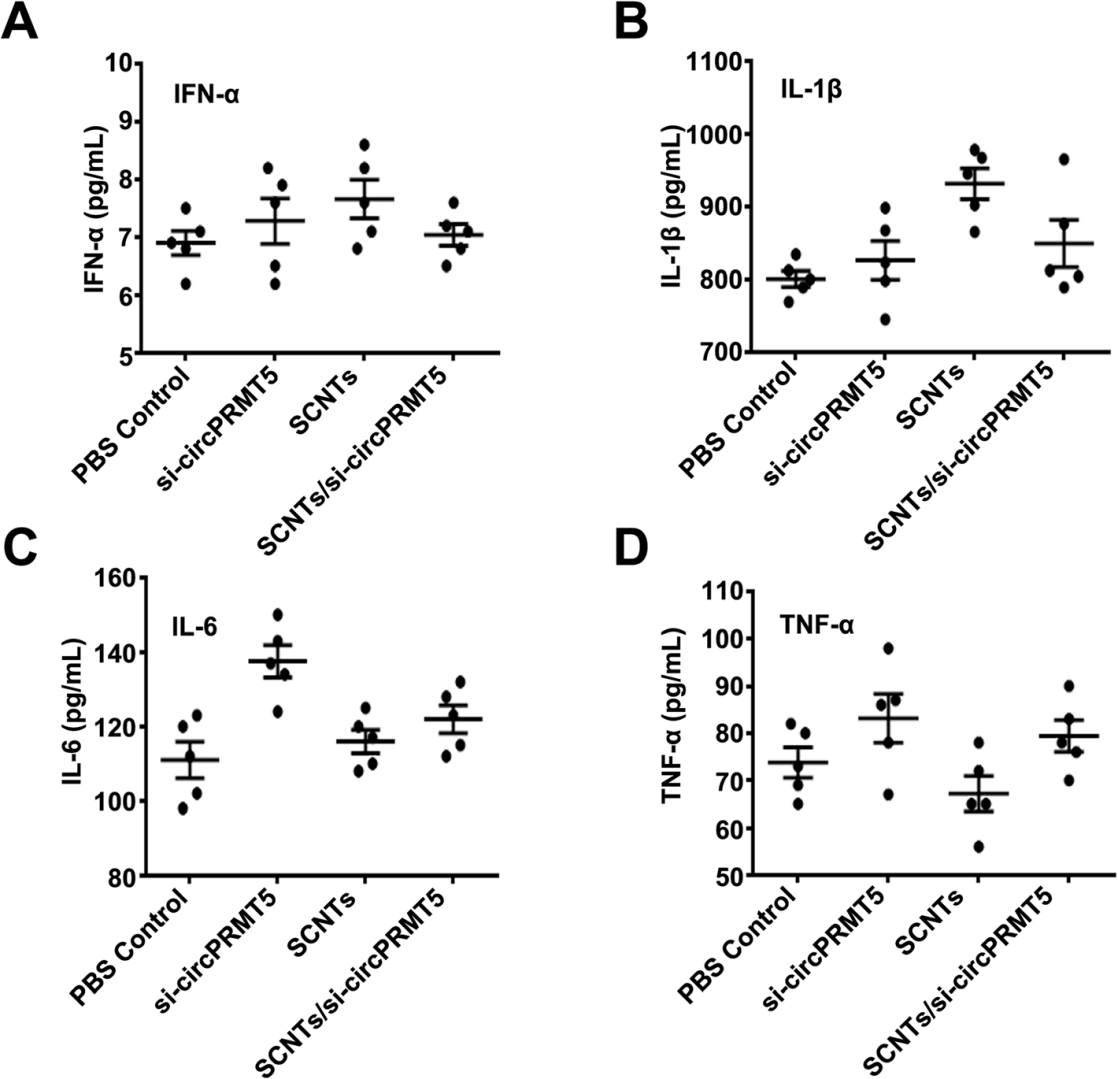
Serum levels of IFN-α (**A**), IL-1β (**B**), interleukin-6 (IL-6) (**C**), and tumor necrosis factor-a (TNF-a) (**D**) at 1 day after intravenous injection into mice (*n* = 5)

SCNTs/si-circPRMT5 were injected into healthy mice via their tail vein to assess their potential tissue toxicity. The mice were killed humanely, and their organs were excised, sectioned, and subjected to H&E staining for histological analysis (Fig. 9). Compared with that in that in the PBS and other groups, in the SCNTs/si-circPRMT5 group, none of the major organs showed marked tissue damage. At the therapeutic dose, SCNTs/si-circPRMT5 generated no toxicity in the kidney or liver, as indicated by the lack of increased in blood serum AST, ALT, TP, TBIL, CREA, and BUN compared with those in the PBS group (Fig. 10). More importantly, compared with those in the PBS and other groups, no physiological differences were noted in the SCNTs/si-circPRMT5 group, according to hematological markers (platelets (PLT), hemoglobin (HGB), red blood cell (RBC), and white blood cell (WBC)). (Fig. 11). Moreover, no significant changes of serum cytokines (such as interleukin-6 (IL-6) and tumor necrosis factor-α (TNF-α), interferon-α (IFN-α), interleukin-1β (IL-1β)) (Fig. 12) were induced. These findings suggested that SCNTs/si-circPRMT5 caused no syndromes, such as hemolytic anemia, or acute infection, and the SCNTs/si-circPRMT5 formulation was well-tolerated in mice.

### Expression levels of circPRMT5/miR-30c/SNAIL1/E-cadherin pathway members in tumor tissues

Next, we analyzed the downstream signaling pathway involved in *circPRMT5*-mediated regulation of bladder cancer cell growth. In our previous study, we reported that *circPRMT5* functions as a prognostic indicator and oncogene in bladder cancer via by the miR-30c/SNAIL1/E-cadherin pathway [12]. Therefore, after sacrifice of the mice, qRT-PCR, western blotting, and IHC analyses of tumor samples were performed. These analyses showed significantly altered levels of members of the miR-30c/SNAIL1/E-cadherin signaling pathway in the tumor mass, which was consistent with the SCNTs-si-circPRMT5-mediated reduction in *circPRMT5* levels (Figs. S5 and S7). By contrast, treatment with SCNTs or free si-circPRMT5 did not alter the miR-30c, SNAIL1, and E-cadherin levels significantly compared with those in the PBS control. Thus, *circPRMT5*-mediated regulation of bladder cancer growth and progression might act through the miR-30c/SNAIL1/E-cadherin signaling pathway.

## Discussion

**Fig. 13:**
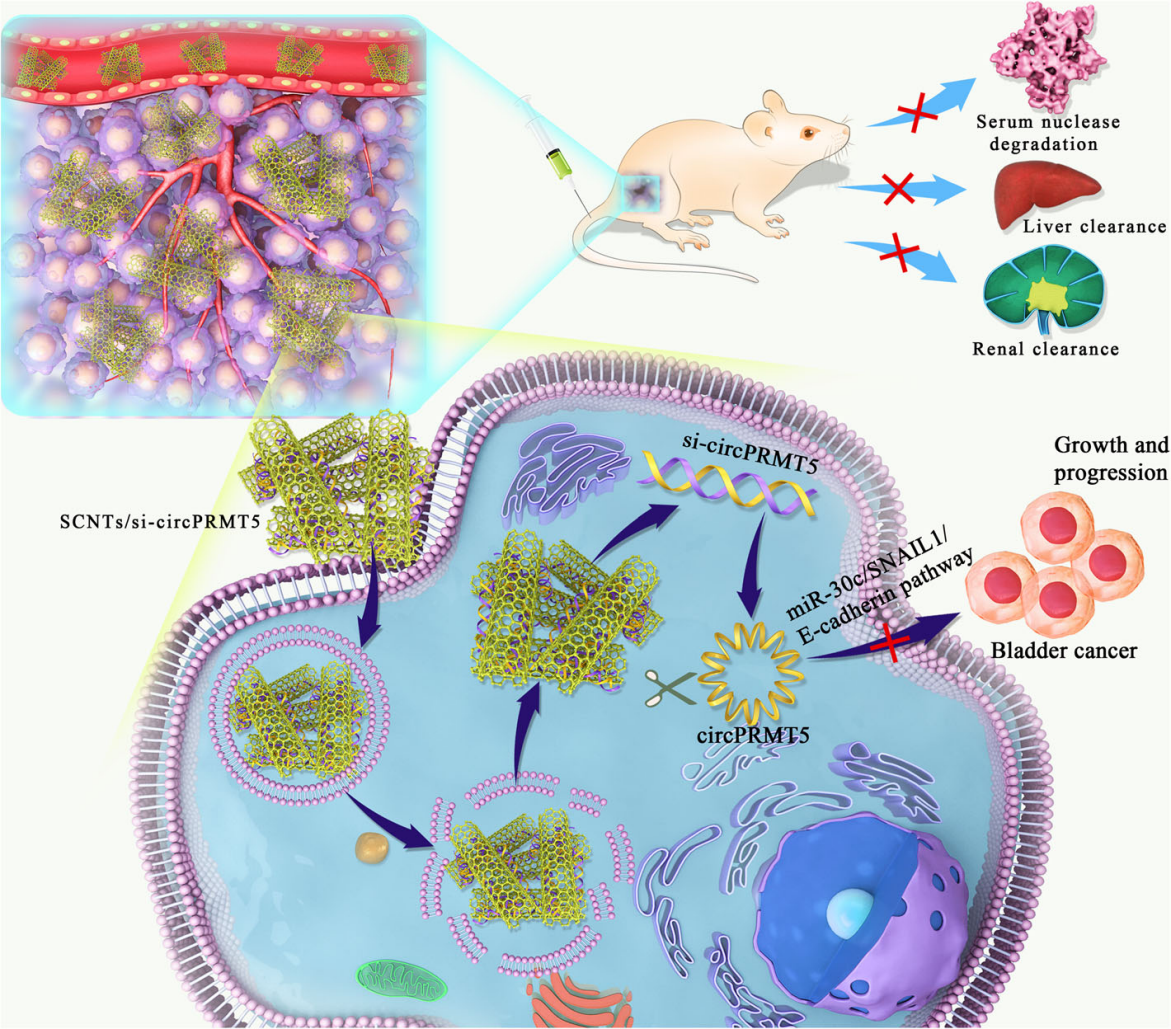
Treatment bladder cancer by SCNTs/si-circPRMT5 nanocomplex. The SCNTs/si-circPRMT5 nanocomplex shields *circPRMT5* siRNA from serum nuclease degradation and liver and renal clearance, thus promoting the accumulation of the *circPRMT5* siRNA in tumor cells, leading to *circPRMT5* silencing in bladder tumors. *circPRMT5* downregulation retards the growth and progression of bladder cancer

Recently, circRNAs have attracted research attention because of their significant functions in various fields of cancer biology [6, 7]. Additionally, circRNAs could contribute to the successful development of individualized medicine to treat cancer [31]. However, the translation of the results of circRNA research to the clinic to regulate circRNA expression in specific human tumor tissues or cells, while avoiding side effects, requires further study. It is necessary to design and deliver molecularly targeted therapeutic circRNAs to cancer areas. Research progress will lead to the discovery of further circRNA physiological and pathological functions. In addition, further circRNA-based therapies and delivery strategies will be formulated, resulting in the development of safe and effective clinical strategies. Since the first discovery of RNAi as a major post-transcriptional gene expression regulatory mechanism [13], it has been suggested as a potential treatment strategy for cancer [32–36]. As far as we know, no studies have reported of the delivery of si-circRNAs and/or anti-circRNAs by nanoparticles into cancer cells. Therefore, we developed SCNT-based nanoparticles to specifically target and deliver si-circPRMT5 into cancer cells. Our findings showed that that this SCNT strategy could effectively deliver si-circPRMT5 to cancer cells. In vivo biodistribution experiments demonstrated that the SCNTs nanoparticles specifically targeted tumors, with little accumulation of their cargo in healthy organs and tissues, representing a significant accomplishment in cancer therapeutics. Furthermore, our data showed that si-circPRMT5 was internalized into bladder cancer cells to effectively silence *circPRMT*5, and regulated its downstream targets (miR-30c, SNAIL1, and E-cadherin) (Fig. 13). Importantly, SCNTs-si-circPRMT5 nanoparticles inhibited bladder cancer development and progression in vitro and in vivo, demonstrating the proof-of-concept that this SCNTs-si-circPRMT5 delivery strategy could be used as a novel and effective antitumor therapy without apparent toxicities or side effects.

SCNTs have been selected as an ideal si-circRNA nanocarrier for facilitate cellular uptake and enhance the activity. SCNTs show that the mixed-charge nanoparticles covered with positively charged external surface and negatively charged internal surface. In neutral condition, SCNTs show negatively charged on its surface and has an electrostatic repulsion to the negatively charged si-circPRMT5. Vacuum impregnation and several times washing is a well-known strategy for achieve the internal loading and remove the external residual, respectively. SCNTs-si-circPRMT5 nanoparticles could reach the tumor sites through the passive target or so called as enhanced permeability and retention (EPR) effect. And further, the nanocarriers could enter tumor cells more effectively than normal cells, because the size parameters (e.g., inner diameter and width) for tumor cells are significantly greater than those of normal cells. It is important that siRNA have been packed into synthetic chrysotile nanotubes, and the size of SCNTs-siRNA complex were not affected. In the end, nanoparticle pharmacokinetics including the degradation and clearance mechanisms is still in its infancy. The clearance and any long-term nanoparticles have been understood from literatures [37].

## Conclusion

In summary, we demonstrated that SCNTs-si-circPRMT5 had a high loading capacity for si-circPRMT5, and induced strong, targeted, and sequence-specific silencing in *circPRMT5*-overexpressing bladder cancer cells. SCNTs-si-circPRMT5 mediated efficient, targeted delivery of si-circPRMT5 to bladder-tumor xenografts, resulting in effective tumor growth suppression, metastasis inhibition, and markedly prolonged survival. As far as we know, this study was the first to develop nanoparticles for the efficient loading and targeted delivery of si-circRNAs or circRNAs. SCNTs-si-circPRMT5 fabrication is safe and easy, making them particularly suitable for clinical translation. SCNTs-si-circRNA nanoparticles represent a versatile, multifunctional, robust, and simple technique to treat cancer via targeted siRNAs.

## Supplementary Information

**Additional file 1.**

## Data Availability

All data generated or analyzed during this study are included in this published article.
