# Erratum to “Treatment of bladder cancer by geoinspired synthetic chrysotile nanocarrier-delivered circPRMT5 siRNA”

**DOI:** 10.34133/bmr.0268

**Published:** 2025-10-28

**Authors:** Chunping Yu, Yi Zhang, Ning Wang, Wensu Wei, Ke Cao, Qun Zhang, Peiying Ma, Dan Xie, Pei Wu, Biao Liu, Jiahao Liu, Wei Xiang, Xing Hu, Xuewen Liu, Jianfei Xie, Jin Tang, Zhi Long, Long Wang, Hongliang Zeng, Jianye Liu

**Affiliations:** ^1^ Department of Urology, The Third Xiangya Hospital of Central South University, No.138, Tongzipo Road, Changsha 410013, Hunan, China.; ^2^ Department of Urology, Sun Yat-sen University Cancer Center, No. 651, Dongfeng Road East, Guangzhou 510060, Guangdong, China.; ^3^ State Key Laboratory of Oncology in Southern China, Collaborative Innovation Center for Cancer Medicine, No. 651, Dongfeng Road East, Guangzhou 510060, Guangdong, China.; ^4^ School of Minerals Processing and Bioengineering, Central South University, No. 932, Lushan South, Changsha 410083, Hunan, China.; ^5^ Department of Onology, The Third Xiangya Hospital of Central South University, No.138, Tongzipo Road, Changsha 410013, Hunan, China.; ^6^ Department of Radiotherapy, The First Affiliated Hospital of Sun Yat-sen University, 58 Zhongshan 2nd Road, Guangzhou 510080, Guangdong, China.; ^7^ Department of Operation Center, The Second Xiangya Hospital of Central South University, People’s Middle Road, Changsha 410008, Hunan, China.; ^8^ Department of Nursing, The Third Xiangya Hospital of Central South University, No.138, Tongzipo Road, Changsha 410013, Hunan, China.; ^9^ Research Institute of Chinese Medicine, Hunan Academy of Chinese Medicine, No.58, Lushan Road, Changsha 410000, Hunan, China.

In the Research Article “Treatment of bladder cancer by geoinspired synthetic chrysotile nanocarrier-delivered *circPRMT5* siRNA,” the authors have discovered an error [1]. The authors found that the hematoxylin and eosin (H&E) staining image of “si-circPRMT5” had an error of overlap with the “SCNTs” in Fig. 8B and C. This error was generated inadvertently during the photography process. All the slides stained with H&E in the experimental groups were placed in a box and managed centrally. Group 8B and 8C were placed closely to each other. In the process of taking and saving pictures, pictures of the other experimental group were mistakenly saved into the wrong group document.

**Fig. 8.**
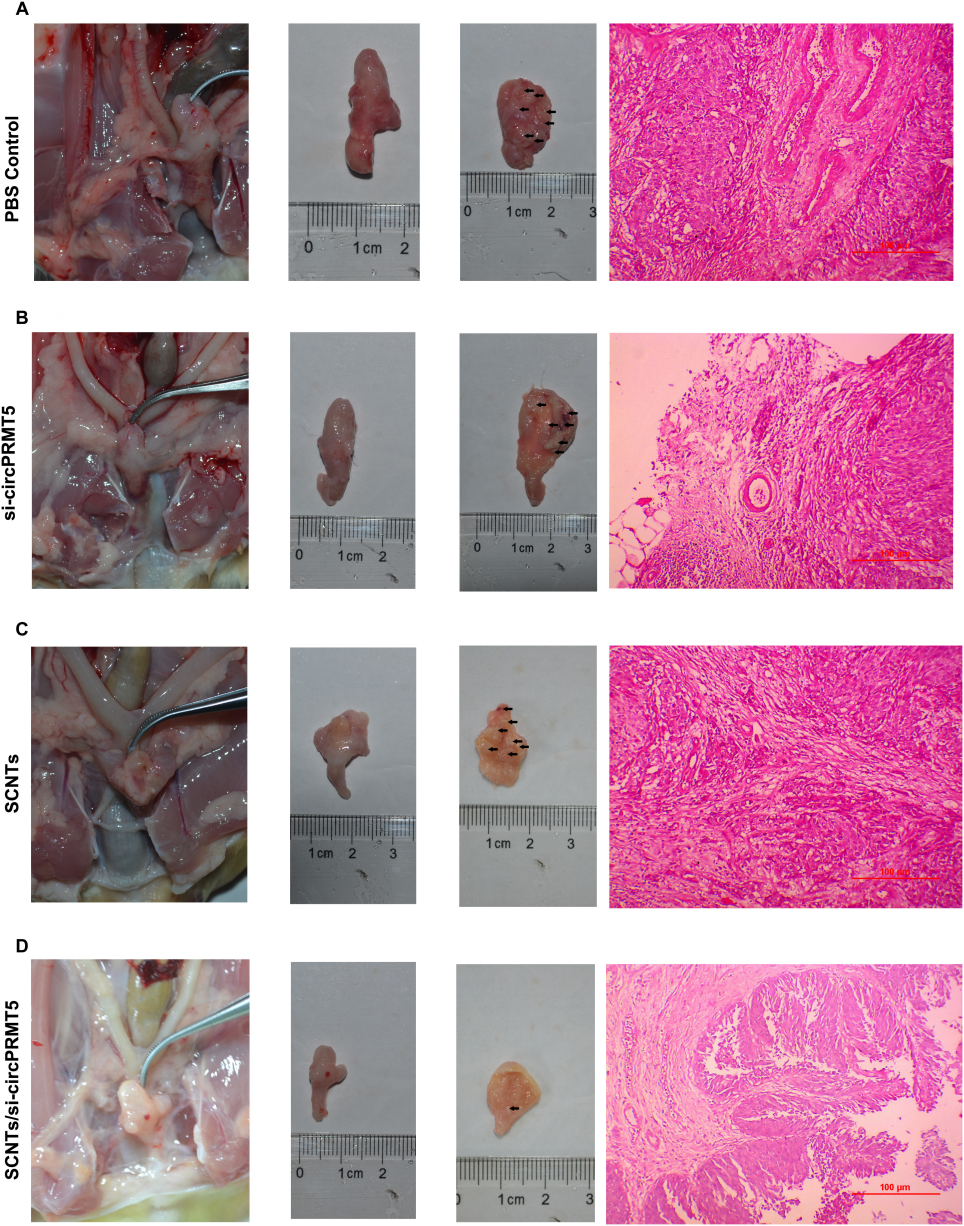
Antitumor effects of SCNTs/si-circPRMT5 in a mouse in situ model of bladder. (A) Images of tissue slices stained with H&E and excised bladders (muscle invasive bladder cancer: ≥ stage pT2) in the PBS-treated group. (B) Images of tissue slices stained with H&E and excised bladders (muscle invasive bladder cancer: ≥ stage pT2) in the si-circPRMT5 treatment group. (C) Images of tissue slices stained with H&E and excised bladders (muscle invasive bladder cancer: ≥ stage pT2) in the SCNT treatment group. (D) Images of tissue slices stained with H&E and excised bladders (noninvasive papillary carcinoma: stage pTa) in the SCNTs/si-circPRMT5 treatment group

The authors apologize for this error. The corrected figure is below, and it has also been updated in the PDF and HTML versions of the article.
