# Supplementary material for: Treatment of bladder cancer by geoinspired synthetic chrysotile nanocarrier-delivered circPRMT5 siRNA
Source: Biomater Res. 2022 Feb 5;26:6. doi: 10.1186/s40824-022-00251-z (PMC8818206; doi:10.1186/s40824-022-00251-z)
Supplement: Supplementary file 1 — Additional file 1. [file 40824_2022_251_MOESM1_ESM.docx]

**Supporting Information**

# Treatment of bladder cancer by geoinspired synthetic chrysotile nanocarrier-delivered *circPRMT5* siRNA

Chunping Yu^1,2,3†^, Yi Zhang^4†^, Ning Wang^2,3†^, Wensu Wei^2,3†^, Ke Cao^5†^, Qun Zhang^6^, Peiying Ma^4^, Dan Xie^3^, Pei Wu^7^, Biao Liu^1^, Jiahao Liu^1^, Wei Xiang^1^, Xing Hu^1^, Xuewen Liu^5^, Jianfei Xie^8^, Jin Tang^1^, Zhi Long^1^, Long Wang^1^, Hongliang Zeng^9^ and Jianye Liu^1*^

^1^Department of Urology, The Third Xiangya Hospital of Central South University, No.138, Tongzipo Road, Changsha, 410013, Hunan, China

^2^Department of Urology, Sun Yat-sen University Cancer Center, No. 651, Dongfeng Road East, Guangzhou, 510060, Guangdong, China

^3^State Key Laboratory of Oncology in Southern China, Collaborative Innovation Center for Cancer Medicine, No. 651, Dongfeng Road East, Guangzhou, 510060, Guangdong, China

^4^School of Minerals Processing and Bioengineering, Central South University, No. 932, Lushan South, Changsha 410083, Hunan, China

^5^Department of Onology, The Third Xiangya Hospital of Central South University, No.138, Tongzipo Road, Changsha, 410013, Hunan, China

^6^Department of Radiotherapy, The First Affiliated Hospital of Sun Yat-sen University, 58 Zhongshan 2nd Road, Guangzhou, 510080, Guangdong, China

^7^Department of Operation Center, The Second Xiangya Hospital of Central South University, People's Middle Road, Changsha, 410008, Hunan, China

^8^Department of Nursing, The Third Xiangya Hospital of Central South University, No.138, Tongzipo Road, Changsha, 410013, Hunan, China

^9^Research Institute of Chinese Medicine, Hunan Academy of Chinese Medicine, No.58, Lushan Road, Changsha, 410000, Hunan, China

^†^These authors contributed equally to this work.

^*^**Corresponding author:** Jianye Liu, Department of Urology, The Third Xiangya Hospital of Central South University, No.138, Tongzipo Road, Changsha, 410013, Hunan, China. Fax: +86 731 88618808. Tel: +86 731 88618828. E-mail: liujianye810@163.com


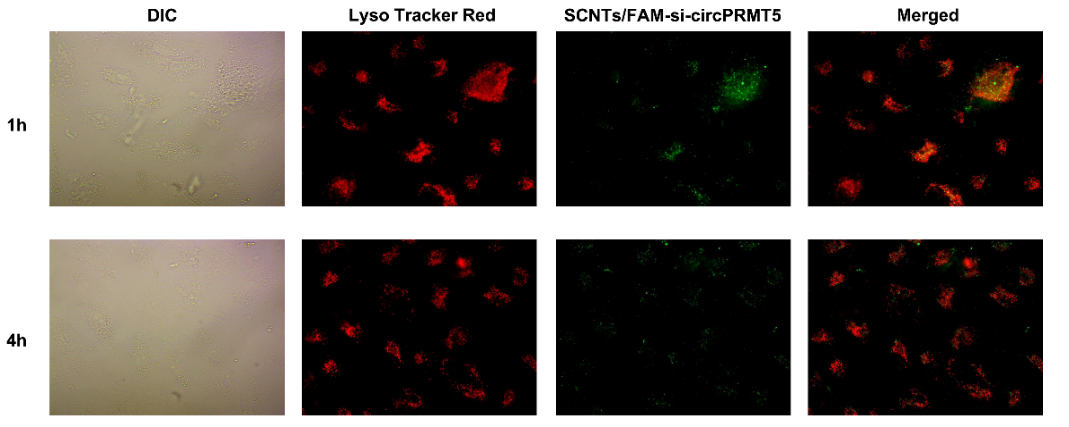


**Fig. S1 Escape of SCNTs/si-circPRMT5 from lysosomes in bladder cancer cells.** Confocal images of T24 cells transfected with SCNTs/si‑circPRMT5 formulations for 1 h at the final siRNA concentration. FAM (green) was used to label si-circPRMT5, lysotracker green (red) was used to stain endosomes/lysosomes, and their merged images are also shown.


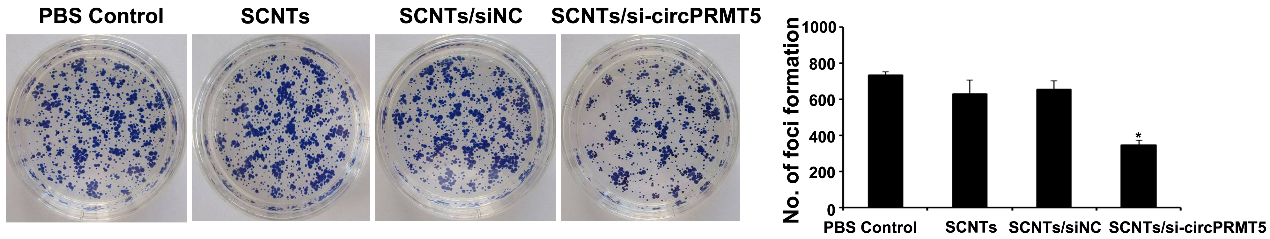


**Fig. S2 Bladder cancer cell colony formation ability after of SCNTs/si-circPRMT5 treatment, assessed via a plate colony assay.** **P* < 0.05.


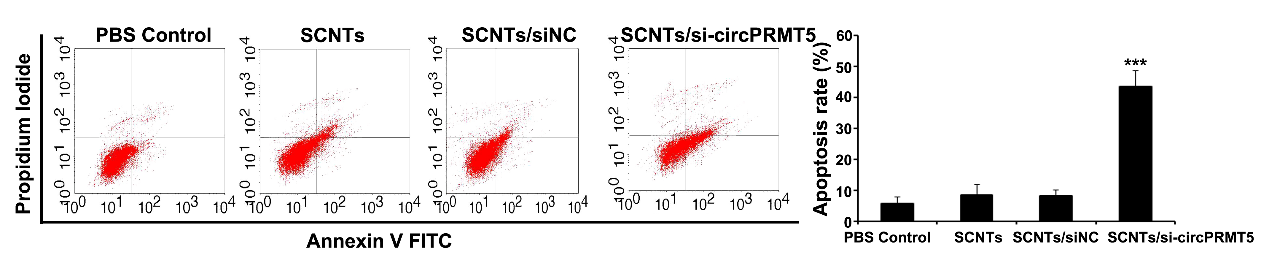


**Fig. S3 *circPRMT5* knockdown by SCNTs/si-circPRMT5 in bladder cancer cells induces apoptosis.** PI and annexin V double-staining to detect apoptotic cells. ****P* < 0.001.


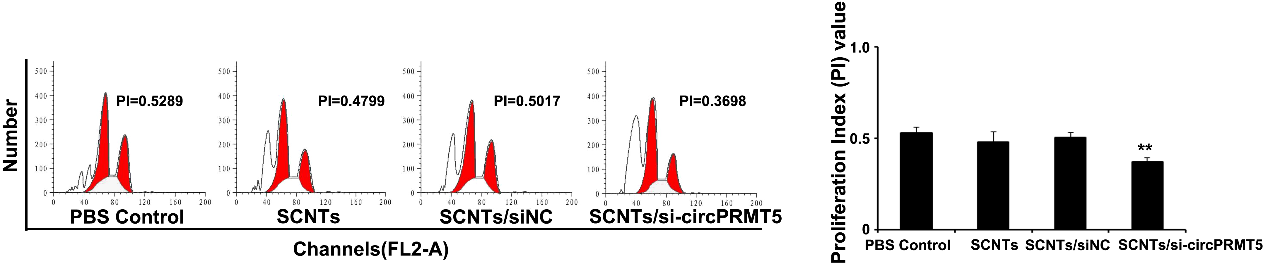


**Fig. S4 *circPRMT5* knockdown by SCNTs/si-circPRMT5 in bladder cancer cells induces S-phase cell cycle arrest.** At 48 h post-treatment, flow cytometry was used to assess the cell cycle distribution. ***P* < 0.01.


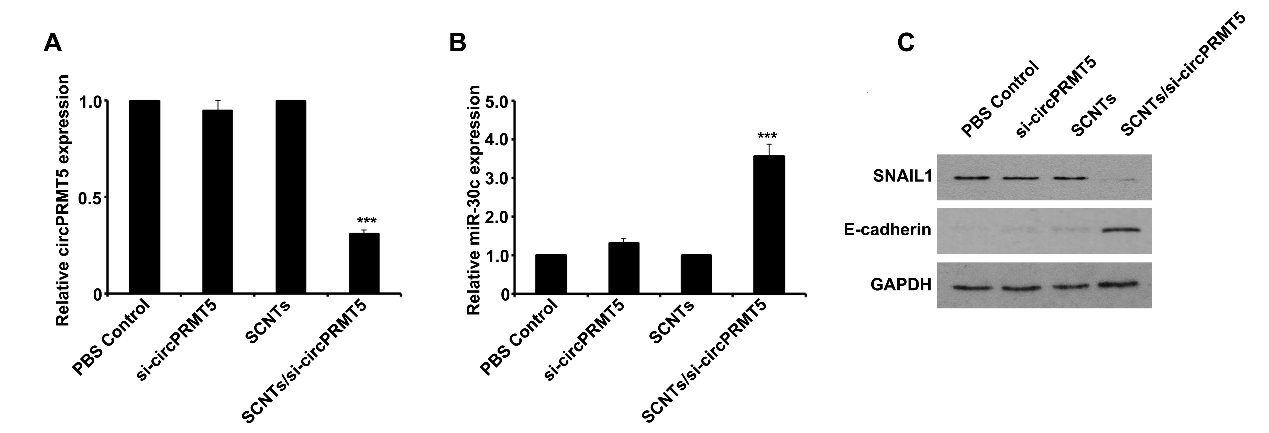


**Fig. S5 qRT-PCR and western blotting analysis of** **the effect of *circPRMT5* silencing by SCNTs/si-circPRMT5 on E-cadherin, SNAIL1, and miR-30c expression in the subcutaneous xenograft model.** ****P* < 0.001.


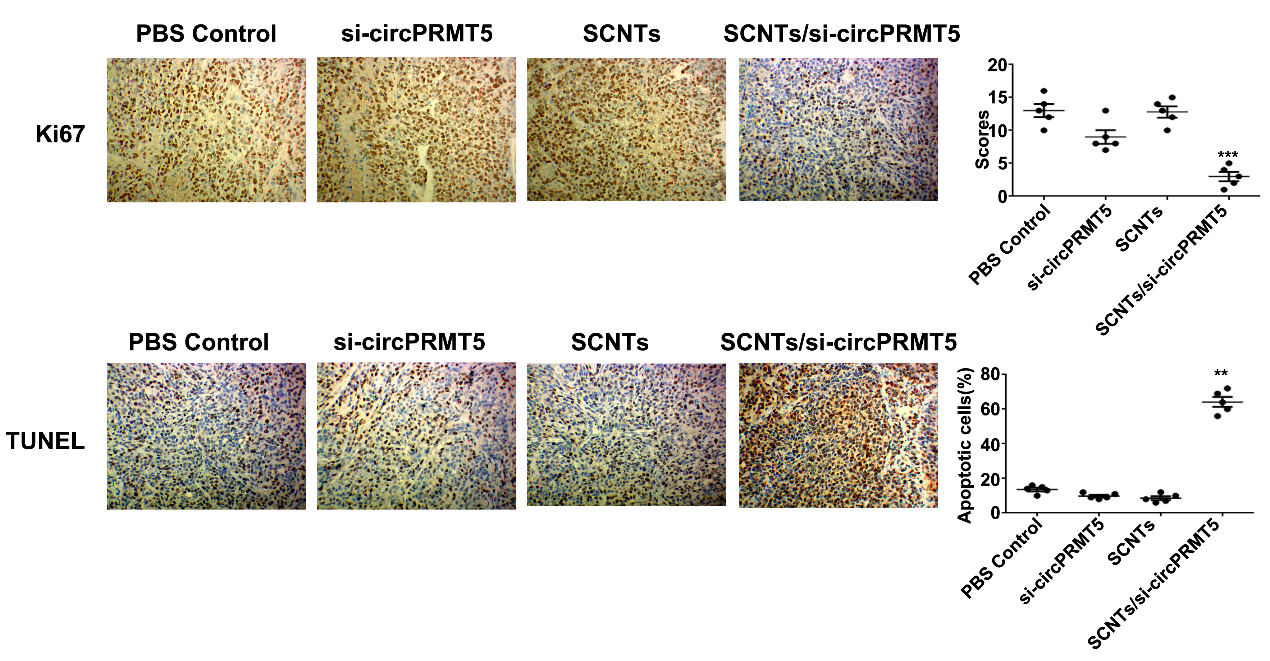


**Fig. S6 Effect of *circPRMT5* silencing by SCNTs/si-circPRMT5 on the expression of Ki67, and TUNEL staining analysis from the subcutaneous xenograft model.** ***P* < 0.01. ****P* < 0.001.


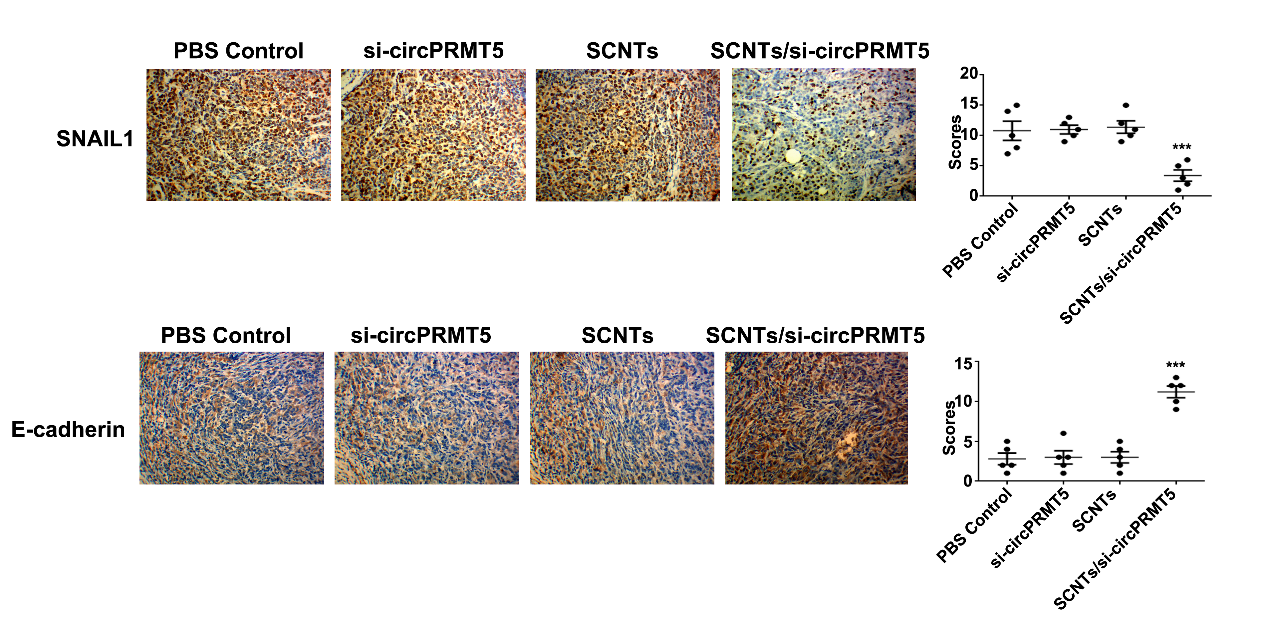


**Fig. S7 IHC analysis of the effect of *circPRMT5* silencing via SCNTs/si-circPRMT5 on E-cadherin and SNAIL1 expression in the subcutaneous xenograft model.** ****P* < 0.001.
